# Contrasting Frustrated Lewis Pair Reactivity with Selenium‐ and Boron‐Based Lewis Acids

**DOI:** 10.1002/anie.201605239

**Published:** 2016-08-03

**Authors:** Lewis C. Wilkins, Benjamin A. R. Günther, Melanie Walther, James R. Lawson, Thomas Wirth, Rebecca L. Melen

**Affiliations:** ^1^ School of ChemistryCardiff UniversityMain Building, Park PlaceCardiffCymru/WalesCF10 3ATUK

**Keywords:** annulation, cyclization, domino reactions, frustrated Lewis pairs, main-group elements

## Abstract

The activation of π‐bonds in diynyl esters has been investigated by using soft and hard Lewis acids. In the case of the soft selenium Lewis acid PhSeCl, sequential activation of the alkyne bonds leads initially to an isocoumarin (1 equiv PhSeCl) and then to a tetracyclic conjugated structure with the isocoumarin subunit fused to a benzoselenopyran (3 equiv PhSeCl). Conversely, the reaction with the hard Lewis acidic borane B(C_6_F_5_)_3_ initiates a cascade reaction to yield a complex π‐conjugated system containing phthalide and indene subunits.

The advent of frustrated Lewis pairs (FLPs) a decade ago has been a major advance in main‐group chemistry, particularly in metal‐free catalysis.^1^ FLP chemistry combines sterically hindered Lewis acids and Lewis bases (Scheme 1, top), where the steric demands of the two components suppress conventional adduct formation and lead to truly unique reactivity. This ever‐expanding field of chemistry now encompasses a vast range of Lewis acid/base combinations which have been shown to effect novel transformations and metal‐free catalytic processes, including small‐molecule activation reactions (e.g. H_2_, N_2_O, CO_2_, CO, SO_2_, alkenes, and alkynes).^2^

**Scheme 1:**
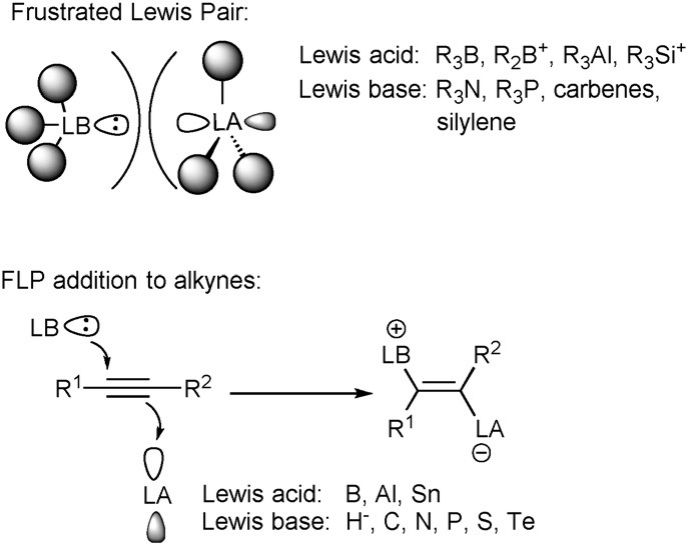
Frustrated Lewis pairs (top) and 1,2‐addition of FLPs to alkynes (bottom).

A particular theme within this area is the cooperative addition of FLPs across C−C π‐bonds (Scheme 1, bottom), which can occur in an intra‐ or intermolecular fashion.^3^ Two different mechanisms for the 1,2‐addition of Lewis acid/base combinations to alkenes and alkynes have been proposed, which either involve concerted addition or initial activation of the π‐bond followed by 1,2‐addition.^4^ In the latter case, π⋅⋅⋅Lewis acid van der Waals interactions have been observed.^4^ However, unlike transition metals, which are able to activate π‐bonds in a synergic fashion through (metal–ligand) bonding and back‐bonding interactions, boron relies solely on the vacant p_*z*_ orbital to activate such unsaturated frameworks. In these reactions, the nucleophilic center can be a wide variety of atoms including hydrogen,^5^ carbon,^6^ nitrogen,^7^ oxygen,^8^ phosphorus,^9^ or sulfur,^10^ while the Lewis acid component has been limited to the first two rows of the p‐block. Typically, hard Lewis acids such as boranes, alanes, or borenium and carbenium cations have been employed.

Other reactivities associated with the use of strong boron Lewis acids involve the synthetically useful carboboration reactions as well as relevant cascade reactions of unsaturated C−C bonds. Recent studies by the Erker and Yamaguchi research groups showcase the use of B(C_6_F_5_)_3_ in a series of rearrangements of aryl‐functionalized diynes to form complex, extensively fused polyaromatic systems such as dibenzopentalenes and derivatives thereof.^11^ The aforementioned activation of alkynes is preeminent in this work, highlighting the fact that, contrary to the traditional concept of HSAB theory, hard Lewis acids are indeed very effective for the activation of soft Lewis bases such as alkynes. It is of note that the synthesis of such polyaromatic systems is seldom described in the literature, with these reports involving rare, noble, or heavy toxic metals such as palladium, gold, or tin.^12^ This provides the impetus to find new synthetic methods to prepare such extended conjugated networks.

Indeed, with a few exceptions,^13^ the use of heavier p‐block elements with FLP‐type reactivity is rarely reported. These heavier p‐block elements are much softer due to their larger atomic radii and lower charge densities, and are more susceptible to polarization, thus potentially offering distinct differences in reactivity compared to their lighter counterparts. Many of these elements are also capable of acting simultaneously as Lewis acids (because of the presence of low‐lying vacant orbitals or σ*‐orbitals) and Lewis bases (because of the presence of lone pairs of electrons when in the *n*−2 or *n*−4 oxidation state). Here we compare the reactivity of methyl‐2‐{[2‐(phenylethynyl)phenyl]ethynyl} benzoate (**1**) with the soft Se‐centered Lewis acid PhSeCl and the hard B‐centered Lewis acid B(C_6_F_5_)_3_ in the pursuit of obtaining novel heterocyclic frameworks such as isocoumarin derivatives. Indeed, isocoumarins are well known for their biological activity and their derivatives have many applications as antimicrobial,^14^ anticancer,^15^ and anti‐inflammatory^16^ agents etc.^17^

The diyne starting material **1** was prepared by a palladium‐catalyzed cross‐coupling reaction according to literature procedures.^18^ The subsequent reactivity of **1** in the presence of excess phenylselenyl chloride (in a 1:3 molar ratio) in CDCl_3_ was monitored by multinuclear NMR spectroscopy. After a few hours at ambient temperature, the ^1^H NMR spectrum showed the formation of a new compound with only aromatic protons, thus indicating the loss of the methyl ester. This observation was confirmed by slow evaporation of the solvent to yield a crop of orange crystals (37 %), which were characterized by single‐crystal X‐ray diffraction and unequivocally confirmed the product to be the salt **2** (Scheme 2, Figure 1). The cationic fragment contains two different selenium environments: 1) a cationic selenonium center and 2) a neutral selane. The counterion is a [PhSeCl_2_]^−^ anion.

**Figure 1:**
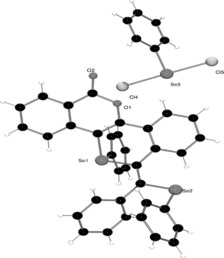
Solid‐state structure of **2**. The chloroform solvate molecule is removed for clarity.

**Scheme 2:**
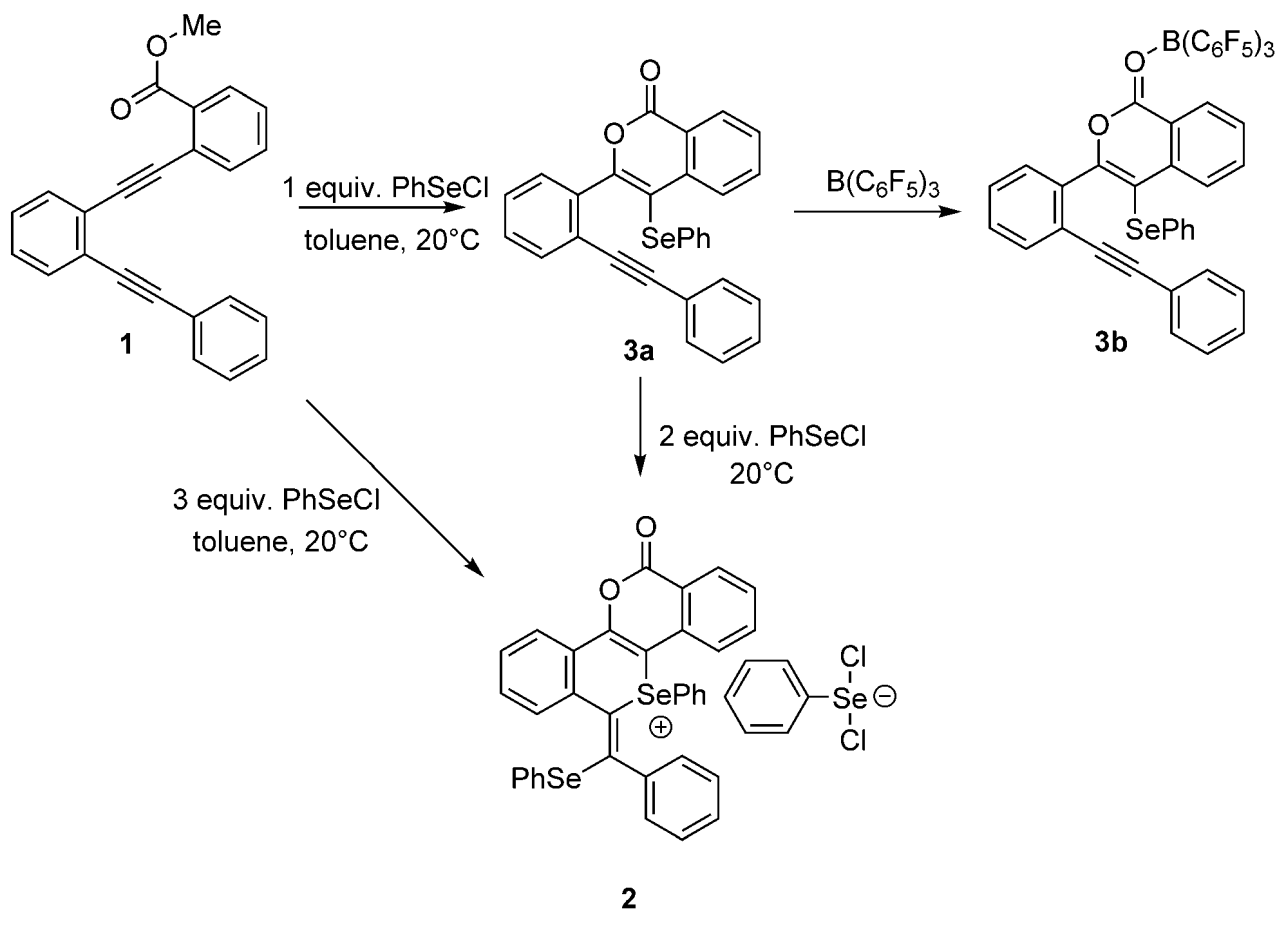
Reactions of **1** with phenylselenyl chloride (PhSeCl) to generate **2** and **3**.

The tetracyclic core of **2** consists of a planar isocoumarin fragment fused to the isoselenochromene as a result of the annulation step. The selenonium cation adopts the expected tetragonal geometry, with C−Se−C bond angles between 96.57(12)° and 100.86(12)° and with Se−C bonds having single bond character (1.912(2)–1.939(3) Å). These observations indicate that the delocalization of the isocoumarin unit does not fully extend throughout the isoselenochromene fragment. Indeed, the lack of planarity across these two moieties further substantiates this observation. The counteranion [PhSeCl_2_]^−^ adopts a T‐shaped geometry with the chlorine atoms occupying the axial positions. The Se−Cl bond lengths (2.445(1) and 2.457(1) Å) and Cl−Se−C bond angles of 90.47(10)° and 90.72(10)° are similar to those described for the only previously reported [PhSeCl_2_]^−^ anion.^19^

The reaction was then performed with 1:1 and 2:1 molar ratios of PhSeCl/**1**. When a 1:1 molar ratio was used, selective activation of the alkynyl functionality proximal to the ester occurred through a 6‐*endo*‐dig cyclization to afford the phenylselenyl‐substituted isocoumarin **3 a** (Scheme 2), which was isolated in 68 % yield. The product was fully characterized by multinuclear NMR spectroscopy and mass spectrometry. However, despite repeated attempts, we were unable to grow single crystals of **3 a** suitable for single‐crystal X‐ray diffraction. The ^77^Se NMR spectrum of compound **3 a** showed a singlet whose chemical shift (*δ*=289.5 ppm) was in agreement with previously reported selanyl compounds.^20^

To probe this reactivity further, multinuclear NMR spectroscopic studies were undertaken to follow the reaction of compound **3 a** with two further equivalents of PhSeCl. These studies revealed ^1^H NMR signals corresponding to the formation of **2**, thus suggesting that **3 a** is an intermediate en route to **2**. A plausible mechanism for the reaction of **1** to **2** via **3 a** is the electrophilic addition of PhSeCl to the alkyne in **1** with formation of a selenirenium cation. This then undergoes an intramolecular ring‐opening reaction by nucleophilic attack from the carbonyl oxygen atom of the ester moiety, thereby resulting in isocoumarin **3 a** with a concomitant loss of chloromethane, as observed in the in situ ^1^H and ^13^C NMR spectra (*δ*=3.05 and 21.5 ppm, respectively).^21^ There is some literature precedent for such selenolactonization reactions.^22^ The reaction of intermediate **3 a** to give the zwitterion **2** may be envisaged as an FLP‐type *trans‐*1,2‐addition of the Lewis basic selane in **3 a** and a second equivalent of the Lewis acidic PhSeCl across the second alkyne (again via the three‐membered selenirenium cation). The chloride ion that is eliminated following the 1,2‐addition is intercepted by the third equivalent of PhSeCl to generate the selenium‐containing counterion [PhSeCl_2_]^−^.

Attempts to promote the second cyclization of **3 a** using the harder Lewis acid B(C_6_F_5_)_3_ failed to lead to the tetracyclic structure reminiscent of **2**. Instead, this afforded the Lewis acid/base adduct (**3 b**, Scheme 2), thus reflecting the hard nature of the lone pair of electrons on the O atom relative to the softer alkyne. Indeed, the structure of the Lewis adduct **3 b** was unambiguously determined by X‐ray diffraction (Figure 2). Such adduct formation has previously been observed using extended‐chain alkynyl benzoates, where adduct formation is also found to be preferred over alkyne activation.^23^Indeed, no evidence for alkyne activation was observed in **3 b** by ^11^B NMR spectroscopy, even after heating in toluene at 100 °C for several days.

**Figure 2:**
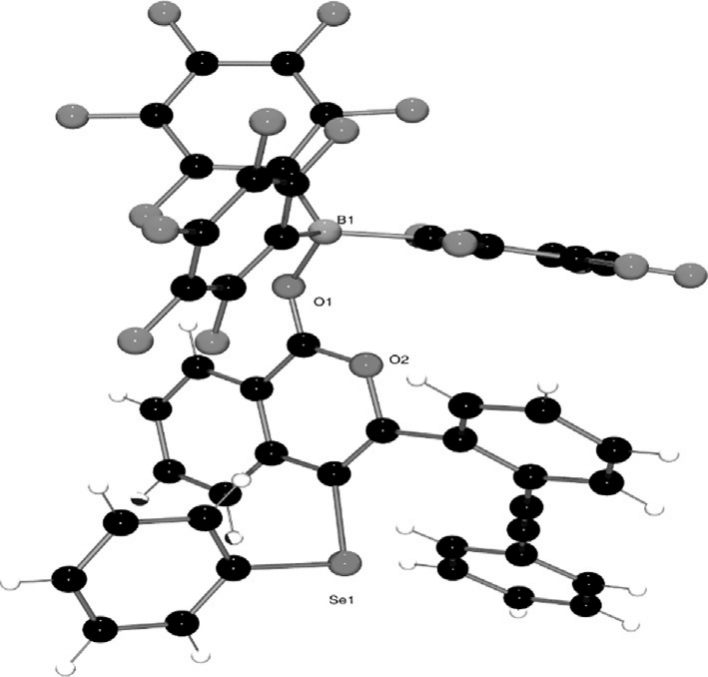
Solid‐state structure of **3 b**.

To compare the Lewis acidity of PhSeCl with more conventional Lewis acids, we investigated the reaction of **1** with B(C_6_F_5_)_3_. The stoichiometric reaction of B(C_6_F_5_)_3_ with **1** (1:1) in toluene resulted in an immediate color change to a dark‐green solution, which afforded block‐shaped red crystals (84 %) upon standing for several hours. Single‐crystal X‐ray diffraction analysis revealed the product to be the conjugated compound **4** (Scheme 3, Figure 3). In this case, an FLP domino reaction occurs in which the sterically encumbered borane activates the less hindered alkyne, thus prompting nucleophilic attack from the more protected alkyne, which is itself attacked by the nucleophilic ester moiety. Interestingly, the formation of **4** from the cyclization of **1** with the Lewis acid B(C_6_F_5_)_3_ is in stark contrast to the formation of **2** from **1** by using PhSeCl. Although bio‐ and biomimetic domino reactions are often acid‐catalyzed, the use of boron compounds in Lewis acid mediated annulation reactions is comparatively rare and remains largely unexplored.^24^, ^25^

**Figure 3:**
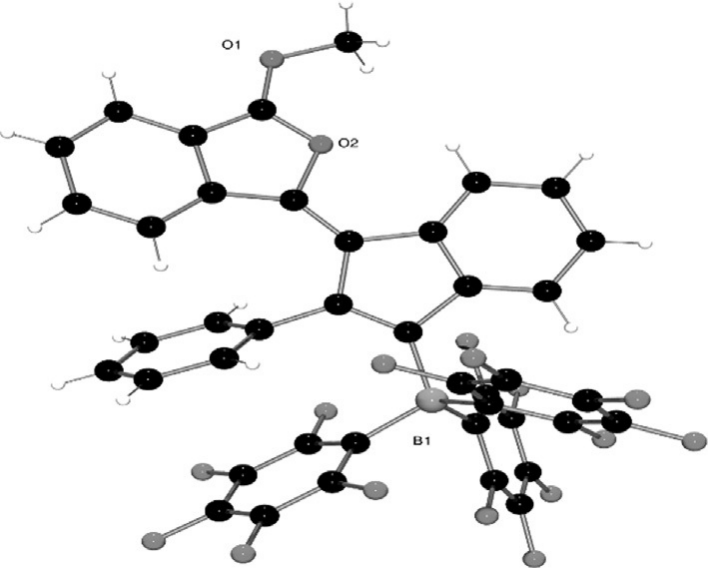
Solid‐state structure of **4**. The disordered toluene solvent is omitted for clarity.

**Scheme 3:**
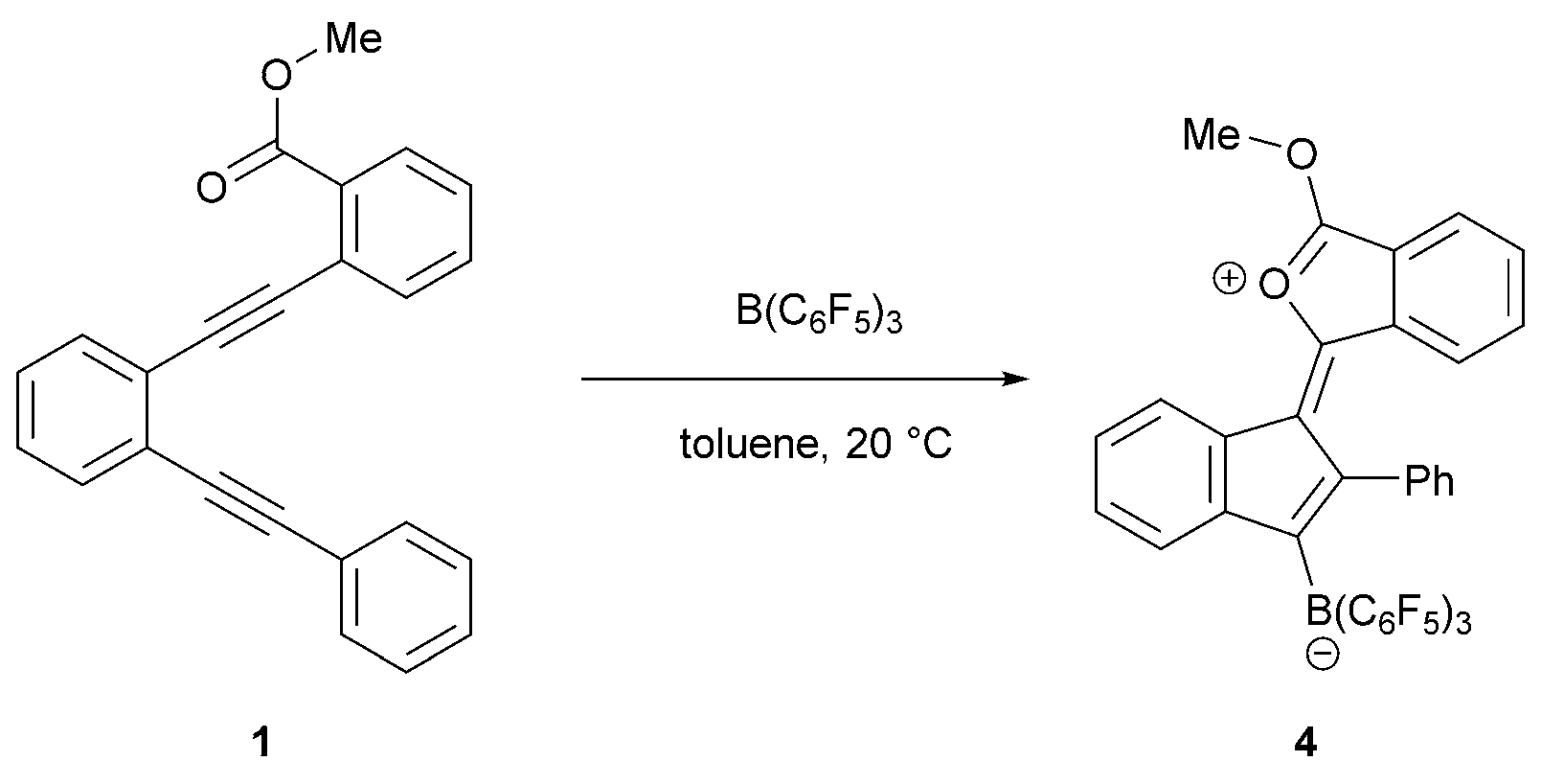
Reaction of **1** with B(C_6_F_5_)_3_.

The structure of **4** consists of an isobenzofuran bicycle connected by a double bond to an indene moiety. Although the isobenzofuran heterocycle and the indene ring are conjugated, they do not lie perfectly in the same plane in the solid state, with the angle between the two rings being 17.99(11)°. The fact that this system does not lie in the same plane clearly reflects the steric constraints in the molecule, which is also revealed in the NMR data. In addition, the phenyl substituent in **4** also rotates out of the plane of the indene ring by 67.87(10)°, thus permitting it to adopt a near coplanar geometry (12.52(9)°) with one of the C_6_F_5_ rings of the B(C_6_F_5_)_3_ moiety with a centroid⋅⋅⋅centroid distance of just 3.4785(16) Å. This value is somewhat shorter than that observed for other C_6_F_5_⋅⋅⋅C_6_H_5_ contacts (3.6421(10) Å).3e

The ^11^B NMR chemical shift for **4** in solution occurs at *δ*=−15.8 ppm as a very sharp singlet, which is synonymous with four‐coordinate vinyl borate species.^3^ Conversely, the ^19^F NMR spectrum is more complex and indicates chemical inequivalence of the three C_6_F_5_ rings, thus suggesting some degree of restricted rotation around the boron–indene bond brought about by the coordination of B(C_6_F_5_)_3_ to the bulky organic fragment in **4**. Three discrete resonances are observed in the ^19^F NMR spectrum for the *para*‐ and *meta*‐fluorine atoms: *δ*=−160.7 ppm (*para*‐F) and *δ*=−165.5 (*meta*‐F). In addition, the *ortho*‐positions within each C_6_F_5_ ring are also inequivalent, thereby resulting in six distinguishable signals between *δ*=−123.0 and −136.1 ppm. This steric hindrance is also observed in the space‐filling model (see the Supporting Information), which clearly shows a very sterically encumbered boron center in which the arene ring of the indene unit interlocks between two C_6_F_5_ rings.

In conclusion, the reactions of soft and hard Lewis acids with dienyl esters show contrasting reactivity that is unprecedented in FLP chemistry. The divergent reactivity of these systems allows novel heterocyclic systems to be generated that incorporate main‐group elements. To date, the addition reactions of FLPs to alkynes have been limited to hard Lewis acids, with the use of softer Lewis acids in FLP‐type chemistry being comparatively rare. Indeed, we are continuing to explore the utility of softer Lewis acids in FLP‐type reactions.

## Supporting information

As a service to our authors and readers, this journal provides supporting information supplied by the authors. Such materials are peer reviewed and may be re‐organized for online delivery, but are not copy‐edited or typeset. Technical support issues arising from supporting information (other than missing files) should be addressed to the authors.

Supplementary
