# Supplementary material for: Contrasting Frustrated Lewis Pair Reactivity with Selenium‐ and Boron‐Based Lewis Acids
Source: Angew Chem Int Ed Engl. 2016 Aug 3;55(37):11292–5. doi: 10.1002/anie.201605239 (PMC5113806; doi:10.1002/anie.201605239)
Supplement: Supplementary file 1 — Supplementary [file ANIE-55-11292-s001.pdf]

Angewandte Chemie
Eine Zeitschrift der Gesellschaft Deutscher Chemiker

Supporting Information

Contrasting Frustrated Lewis Pair Reactivity with Selenium- and Boron-Based Lewis Acids

*Lewis C. Wilkins, Benjamin A. R. Günther, Melanie Walther, James R. Lawson, Thomas Wirth, and Rebecca L. Melen**

anie_201605239_sm_miscellaneous_information.pdf

Table of Contents

1. Experimental section **2**

1.1 General considerations 2

1.2 Synthesis of starting materials 2

1.2.1 Synthesis of methyl 2-((trimethylsilyl)ethynyl)benzoate 2

1.2.2 Synthesis of methyl 2-ethynylbenzoate 3

1.2.3 Synthesis of 1-bromo-2-(phenylethynyl)benzene 3

1.2.4 Synthesis of methyl-2-((2-(phenylethynyl)phenyl)ethynyl)benzoate (**1**) 4

1.3 Synthesis of products 4

1.3.1 Synthesis of **2** 4

1.3.2 Synthesis of **3a** 5

1.3.3 Synthesis of **3b** 5

1.3.4 Synthesis of **4** 6

2. NMR spectra **7**

2.1 NMR spectra of starting materials 7

2.2 NMR spectra of products 11

3. X-ray crystallography **26**

3.1 Table of crystallographic details 26

3.2 Crystal structures 27

4. References **31**

1. Experimental section

1.1 General considerations

With the exception of the synthesis of starting materials, all reactions and manipulations were carried out under an atmosphere of dry, O_2-free nitrogen using standard double-manifold techniques with a rotary oil pump. A nitrogen-filled glove box (MBRAUN) was used to manipulate solids including storage of the starting materials, room temperature reactions, product recovery and sample preparation for analysis. Molecular sieves (4 Å) were dried at 150 °C for 48 h prior to use. Toluene, THF, hexane and CH_2Cl_2 solvents were dried by employing a Grubbs-type column system (MBRAUN), degassed and stored over molecular sieves under a nitrogen atmosphere. Deuterated solvents ($CDCl_3$ and CD_2Cl_2) were dried over molecular sieves before use. Chemicals were purchased from commercial suppliers and used as received. Starting material methyl-2-((2-(phenylethynyl)phenyl)ethynyl)benzoate (**1**) was prepared according to literature methods.[1] ^{1}H, ^{13}C, ^{11}B, ^{19}F and ^{77}Se spectra were recorded on a Bruker Avance DPX-500, Bruker Avance 400, or Jeol Eclipse 300 spectrometer. Chemical shifts are expressed as parts per million (ppm, δ) downfield of tetramethylsilane (TMS) (δ = 0 ppm) and are referenced to $CDCl_3/CD_2Cl_2$ as internal standards. NMR spectra were referenced to $CFCl_3$ (^{19}F), $BF_3{\cdot}Et_2O/CDCl_3$ (^{11}B) and Me_2Se (^{77}Se). The description of signals include: s = singlet, d = doublet, t = triplet, m = multiplet and br. = broad. All coupling constants are absolute values and are expressed in Hertz (Hz). ^{13}C NMR spectra were measured as ^{1}H decoupled. Yields are given as isolated yields. All spectra were analyzed assuming a first order approximation. IR Spectra were recorded using a Shimadzu IRAffinity-1 spectrophotometer. The description of signals include w = weak, s = strong, br. = broad. Mass spectrometry was carried out in house using a Waters LCT Premier/XE or a Waters GCT Premier spectrometer and were calculated using ^{10}B. Melting points were measured in open glass capillary tubes in a Büchi melting point apparatus. We acknowledge Prof. Dr. A. S. K. Hashmi for an authentic sample of compound **1** and W. R. Grace for the kind donation of $B(C_6F_5)_3$.

1.2 Synthesis of starting materials

1.2.1 Synthesis of methyl 2-((trimethylsilyl)ethynyl)benzoate

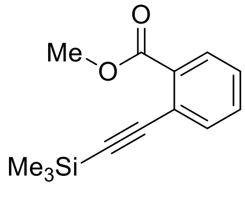

Methyl 2-((trimethylsilyl)ethynyl)benzoate was prepared according to literature methods.[1] To a solution of methyl 2-iodobenzoate (2.52 g, 9.60 mmol), copper iodide (73.3 mg, 385 μmol) and $PdCl_2(PPh_3)_2$ (135 mg, 193 μmol) in THF (25 ml) was added triethylamine (2.67 ml, 19.3 mmol). A solution of ethynyltrimethylsilane (1.42 g, 14.5 mmol) in THF (5 ml) was added dropwise and the reaction mixture was stirred for 19 h at 23 °C. The mixture was filtered through Celite and diluted with water (150 ml). The phases were separated and the aqueous layer was extracted with diethyl ether (3 x 50 ml). The combined organic layers were

washed with aq. NaCl solution (3 x 50 ml) and dried over $MgSO_4$. The solvent was removed *in vacuo* yielding methyl 2-((trimethylsilyl)ethynyl)benzoate as a brown oil (2.13 g, 9.18 mmol, 95%). The NMR data is in agreement with that reported in the literature.[1] **^{1}H NMR** (500 MHz, $CDCl_3$, 298 K): 7.90 (dd, $^3J_{HH}$ = 7.7 Hz, $^4J_{HH}$ = 1.2 Hz, 1H), 7.58 (dd, $^3J_{HH}$ = 7.7 Hz, $^4J_{HH}$ = 1.2 Hz, 1H), 7.44 (td, $^3J_{HH}$ = 7.7 Hz, $^4J_{HH}$ = 1.2 Hz, 1H), 7.37 (td, $^3J_{HH}$ = 7.7 Hz, $^4J_{HH}$ = 1.2 Hz, 1H), 3.92 (s, 3H), 0.27 (s, 9H).

1.2.2 Synthesis of methyl 2-ethynylbenzoate

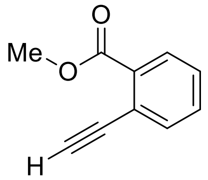

Methyl 2-ethynylbenzoate was prepared according to literature methods.[1] To a solution of methyl 2-((trimethylsilyl)ethynyl)benzoate (2.13 g, 9.18 mmol) in MeOH (20 ml) and CH_2Cl_2 (10 ml) was added K_2CO_3 (3.86 g, 27.60 mmol). The reaction mixture was stirred for 13 h at 23 °C, upon completion the reaction mixture was diluted with water (50 ml). The layers were separated and the aqueous phase was extracted with diethyl ether (3 x 30 ml). The combined organic layers were washed with saturated aq. NaCl solution (3 x 30 ml) and dried over $MgSO_4$. The solvent was removed *in vacuo* yielding methyl 2-ethynylbenzoate as a brown oil (1.19 g, 7.40 mmol, 81%). The NMR data is in agreement with that reported in the literature.[1] **^{1}H NMR** (500 MHz, $CDCl_3$, 298 K): 7.94 (dd, $^3J_{HH}$ = 7.8 Hz, $^4J_{HH}$ = 1.1 Hz, 1H), 7.63 (dd, $^3J_{HH}$ = 7.8 Hz, $^4J_{HH}$ = 1.1 Hz, 1H), 7.48 (td, $^3J_{HH}$ = 7.7 Hz, $^4J_{HH}$ = 1.4 Hz, 1H), 7.41 (td, $^3J_{HH}$ = 7.7 Hz, $^4J_{HH}$ = 1.3 Hz, 1H), 3.93 (s, 3H), 3.40 (s, 1H).

1.2.3 Synthesis of 1-bromo-2-(phenylethynyl)benzene

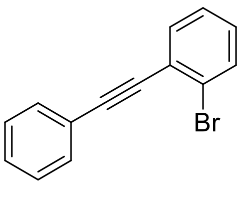

1-bromo-2-(phenylethynyl)benzene was prepared according to literature methods.[1] To a solution of 1-bromo-2-iodobenzene (1.40 g, 4.93 mmol), copper iodide (37.6 mg, 197 μmol) and $PdCl_2(PPh_3)_2$ (69.2 mg, 98.6 μmol) in THF (15 ml) was added triethylamine (1.37 ml, 9.86 mmol). A solution of phenylacetylene (756 mg, 7.40 mmol) in THF (3 ml) was added dropwise and the reaction mixture was stirred for 16 h at 23 °C. The mixture was filtered through Celite and diluted with water (150 ml). The phases were separated and the aqueous layer was extracted with diethyl ether (3 x 50 ml). The combined organic layers were washed with saturated aq. NaCl solution (3 x 50 ml) and dried over $MgSO_4$. The solvent was removed *in vacuo* with the crude oil being purified by column chromatography (silica, eluent: *n*-hexane), yielding 1-bromo-2-(phenylethynyl)benzene as a brown oil (0.83 g, 3.20 mmol, 65%). The NMR data is in agreement with that reported in the literature.[1] **^{1}H NMR** (500 MHz, $CDCl_3$, 298 K): = 7.52 – 7.63 (m, 4H), 7.36 – 7.37 (m, 3H), 7.30 (td, $^3J_{HH}$ = 7.4 Hz, $^4J_{HH}$ = 1.2 Hz, 1H), 7.18 (td, $^3J_{HH}$ = 7.8 Hz, $^4J_{HH}$ = 1.6 Hz, 1H).

1.2.4 Synthesis of methyl-2-((2-(phenylethynyl)phenyl)ethynyl)benzoate (**1**)

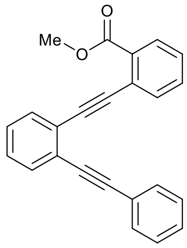

Methyl-2-((2-(phenylethynyl)phenyl)ethynyl)benzoate (**1**) was prepared according to literature methods.[1] To a solution of 1-bromo-2-(phenylethynyl)benzene (829 mg, 3.22 mmol), copper iodide (12 mg, 64.4 μmol), $Pd(PPh_3)_4$ (74 mg, 64.4 μmol) and *n*-butylamine (1.29 ml, 13.0 mmol) in dry diethyl ether (10 ml) was added methyl 2-ethynylbenzoate (516 mg, 3.22 mmol). The resulting mixture was heated to 60 °C and stirred for 17 h. After cooling to rt, the mixture was quenched with saturated aq. NH_4Cl solution (20 ml). The layers were separated and the aqueous layer was extracted with diethyl ether (3 x 10 ml). The combined organic layers were dried over $MgSO_4$, filtered and concentrated *in vacuo*. The crude oil was purified by column chromatography to give **1** (396 mg, 0.97 mmol, 30%). The NMR data is in agreement with that reported in the literature.[1] **^{1}H NMR** (500 MHz, $CDCl_3$, 298 K): 7.99 (dd, $^{3}J_{HH}$ = 7.9 Hz, $^{4}J_{HH}$ = 1.4 Hz, 1H), 7.71 (dd, $^{3}J_{HH}$ = 7.7 Hz, $^{4}J_{HH}$ = 1.4 Hz, 1H), 7.61 – 7.63 (m, 1H), 7.55 – 7.59 (m, 3H), 7.47 (td, $^{3}J_{HH}$ = 7.6 Hz, $^{4}J_{HH}$ = 1.4 Hz, 1H), 7.39 (td, $^{3}J_{HH}$ = 7.8 Hz, $^{4}J_{HH}$ = 1.4 Hz, 1H), 7.32 – 7.35 (m, 5H), 3.89 (s, 3H).

1.3 Synthesis of products

1.3.1 Synthesis of (E)-11-phenyl-12-(phenyl(phenylselanyl)methylene)-11,12-dihydro-6H-11l3-isoselenochromeno[4,3-c]isochromen-6-one dichlorophenylselanate (**2**)

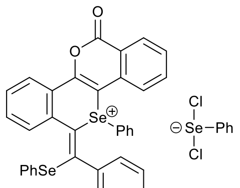

A solution of phenylselenyl chloride (29.1 mg, 150 µmol, 3 equiv.) in $CDCl_3$ (0.5 ml) was added to methyl-2((2-phenylethynyl) phenylethynyl)benzoate (17 mg, 50.0 µmol, 1 equiv.). Slow evaporation of the solvent yielded orange crystals which could be characterized by single crystal X-ray diffraction. The crystals were washed with *n*-hexane (2 x 3 ml) and dried *in vacuo* to give the pure product (16 mg, 18.6 µmol, 37%). **mp**. = 128 – 133 °C. **^{1}H NMR** (400 MHz, CD_2Cl_2, 298 K): 8.39 (d, $^{3}J_{HH}$ = 7.8 Hz, 1H), 8.16 (dd, $^{3}J_{HH}$ = 7.0 Hz, $^{4}J_{HH}$ = 1.4 Hz, 1H), 8.09 (dd, $^{3}J_{HH}$ = 7.3 Hz, $^{4}J_{HH}$ = 1.8 Hz, 1H), 7.84 (t, $^{3}J_{HH}$ = 7.3 Hz, 1H), 7.77 (d, $^{3}J_{HH}$ = 7.8 Hz, 2H), 7.69 (t, $^{3}J_{HH}$ = 7.5 Hz, 1H), 7.59 – 7.56 (m, 2H), 7.54 – 7.38 (m, 11H), 7.30 (d, $^{3}J_{HH}$ = 7.3 Hz, 2H), 7.21 – 7.20 (m, 4H), 7.06 (t, $^{3}J_{HH}$ = 7.6 Hz, 2H). **^{13}C{^{1}H} NMR** (101 MHz, CD_2Cl_2, 298 K): 163.5 (s), 159.2 (s), 153.3 (s), 137.8 (s), 137.5 (s), 136.6 (s), 136.3 (br. s), 134.0 (s), 133.3 (s), 133.0 (s), 132.1 (s), 132.0 (s), 132.0 (s), 131.9 (s), 131.8 (s), 131.5 (s), 130.4 (s), 130.3 (s), 130.1 (s), 130.1 (s), 129.8 (s), 129.9 (s), 129.7 (s), 129.7 (s), 129.6 (s), 129.0 (br. s), 128.6 (br. s), 127.9 (s), 127.9 (s), 127.7 (s), 127.4 (s), 124.7 (s), 120.8 (s), 114.1 (s), 104.9 (s). **IR (ATR)**: *v* = 3052 (w), 1263 (s), 1263 (s), 895 (w), 746 (s), 702 (s) cm^{-1}. **HRMS** (ES^{+}) *m/z* calculated for $[C_{35}H_{23}O_2Se_2]^+$ $[M-PhSeCl_2]^+$: 635.0028; Found 635.0037. **Elemental analysis** (%) calculated, C: 57.62 H: 3.57. Found, C: 57.56 H: 3.43. Note: Sparse solubility of selenocyclic salt **2** resulted in a very weak ^{77}Se NMR spectrum. Trials in many different deuterated solvents did not lead to a sample sufficiently concentrated enough to carry out this spectroscopic method.

1.3.2 Synthesis of 3-(2-(phenylethynyl)phenyl)-4-(phenylselanyl)-1H-isochromen-1-one (**3a**)

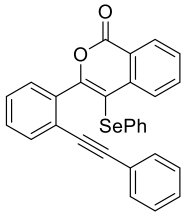

A solution of phenylselenyl chloride (38 mg, 0.2 mmol) in toluene (1 ml) was added to methyl-2((2-phenylethynyl)phenylethynyl)benzoate (**1**) (67 mg, 0.2 mmol) and the reaction left overnight at room temperature. The solvent was then removed and the crude product washed with hot *n*-hexane. Drying *in vacuo* afforded the pure product (65 mg, 0.14 mmol, 68%). **^{1}H NMR** (500 MHz, $CDCl_3$, 298 K): 8.39 (dd, $^{3}J_{HH}$ = 7.7 Hz, $^{4}J_{HH}$ = 1.0 Hz, 1H), 8.05 (d, $^{3}J_{HH}$ = 8.0 Hz, 1H), 7.71 (td, $^{3}J_{HH}$ = 8.0 Hz, $^{4}J_{HH}$ = 1.3 Hz, 1H), 7.64 (dd, $^{3}J_{HH}$ = 7.7 Hz, $^{4}J_{HH}$ = 1.0 Hz, 1H), 7.55 (t, $^{3}J_{HH}$ = 7.7 Hz, 1H), 7.51 (dd, $^{3}J_{HH}$ = 7.4 Hz, $^{4}J_{HH}$ = 1.0 Hz, 1H), 7.45 (td, $^{3}J_{HH}$ =7.6 Hz, $^{4}J_{HH}$ = 1.6 Hz, 1H), 7.39 (td, $^{3}J_{HH}$ = 8.0 Hz, $^{4}J_{HH}$ = 1.2 Hz, 1H), 7.33 – 7.31 (m, 2H), 7.27 – 7.22 (m, 5H), 7.09 – 7.04 (m, 3H). **^{13}C{^{1}H} NMR** (126 MHz, $CDCl_3$, 298 K): 161.8 (s), 158.5 (s), 138.0 (s), 137.9 (s), 136.7 (s), 135.3 (s), 132.0 (s), 131.6 (s), 131.4 (s), 129.9 (s), 129.9 (s), 129.8 (s), 129.7 (s), 129.1 (s), 128.8 (s), 128.5 (s), 128.3 (s), 128.2 (s), 128.1 (s), 127.9 (s), 126.6 (s), 125.3 (s), 123.9 (s), 122.8 (s), 121.2 (s), 107.7 (s), 94.3 (s), 87.4 (s). **^{77}Se NMR** (95 MHz, $CDCl_3$, 298 K): 289.5. **HRMS** (ES^{+}) *m/z* calculated $[C_{29}H_{18}O_2Se]^{+}$ $[M]^{+}$: 478.0472; found: 478.0475. The following elemental analysis was found to be the most representative of three separate attempts. **Elemental analysis** (%) calculated, C: 72.96 H: 3.80. Found, C: 71.48 H: 3.41.

1.3.3 Synthesis of 3-(2-(phenylethynyl)phenyl)-4-(phenylselanyl)-1H-isochromen-1-one tris(perfluorophenyl) borane adduct (**3b**)

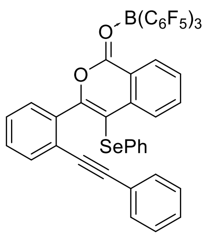

A solution of phenylselenyl chloride (19 mg, 0.1 mmol) in $CDCl_3$ (0.5 ml) was added to methyl-2((2-phenylethynyl)phenylethynyl)benzoate (**1**) (34 mg, 0.1 mmol). After 3 h tris(pentafluorophenyl)borane (51 mg, 0.1 mmol) was added which was accompanied by a color change from an orange solution to dark red/black. The reaction mixture was heated to 40 °C for 3 d. The dark red solution was concentrated *in vacuo*, washed with *n*-hexane and concentrated again *in vacuo*. Recrystallisation from CH_2Cl_2 and *n*-hexane yielded the product as red-brown crystals which could be characterized by single crystal X-ray diffraction (41 mg, 0.4 mmol, 41%). **^{1}H NMR** (500 MHz, $CDCl_3$, 298 K): 8.60 (d, $^{3}J_{HH}$ = 8.2 Hz, 1H), 8.23 (d, $^{3}J_{HH}$ = 8.2 Hz, 1H), 8.00 (t, $^{3}J_{HH}$ = 7.7 Hz, 1H), 7.79 (t, $^{3}J_{HH}$ = 7.7 Hz, 1H), 7.54 (d, $^{3}J_{HH}$ = 7.2 Hz, 1H), 7.45 (t, $^{3}J_{HH}$ = 8.2 Hz, 1H), 7.29 (t, $^{3}J_{HH}$ = 7.7 Hz, 1H), 7.20 (t, $^{3}J_{HH}$ = 8.2 Hz, 1H), 6.92 – 7.15 (m, 10H). **^{13}C{^{1}H} NMR** partial (126 MHz, $CDCl_3$, 298 K): 170.2 (s), 156.4 (s), 148.0 (m), 140.0 (s), 139.9 (s), 137.3 (m), 133.0 (s), 132.4 (s), 131.5 (s), 131.2 (s), 131.1 (s), 129.8 (s), 129.4 (s), 129.2 (s), 129.1 (s), 129.1 (s), 128.5 (s), 128.4 (s), 128.0 (s), 124.0 (s), 121.9 (s), 117.7 (s), 116.5 (m), 95.1 (s), 85.7 (s). **^{11}B NMR** (160 MHz, $CDCl_3$, 298 K): -0.51 (br. s). **^{19}F NMR** (283 MHz, $CDCl_3$, 298 K): - 135.0 (d, $^{3}J_{FF}$ = 20.5 Hz, 6F, *o*-F), - 157.3 (t, $^{3}J_{FF}$ = 18.8 Hz, 3F, *p*-F), - 163.8 (t, $^{3}J_{FF}$ = 21.7 Hz, 6F, *m*-F). **^{77}Se NMR** (95 MHz, $CDCl_3$, 298 K): 289.4 (s). **IR (ATR)**: v = 2916 (w, br.), 1493 (m), 725 (s), 698 (s) cm^{-1}. **HRMS** (ES^{+}) *m/z* calculated for $[C_{47}H_{18}BF_{15}O_2Se]^{+}$ $[M]^{+}$: 990.0326; Found: 990.0338. **Elemental analysis** (%) calculated, C: 57.06, H: 1.83. Found, C: 57.15, H: 1.79.

1.3.4 Synthesis of (E)-(1-(3-methoxy-1H-isobenzofuran-2-ium-1-ylidene)-2-phenyl-1H-inden-3-yl)tris(perfluorophenyl)borate (**4**)

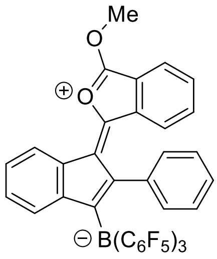

Methyl-2-((2-(phenylethynyl)phenyl)ethynyl)benzoate (**1**) (67 mg, 0.2 mmol) and $B(C_6F_5)_3$ (102 mg, 0.2 mmol) were dissolved in toluene (1 ml) to give a yellow solution, which quickly changed color to a very dark green. The reaction mixture was left at room temperature for 4 h. During this time crystals were formed, which were suitable for X-ray diffraction. The solvent was evaporated and the remaining crystals were washed with hexane (6 x 2 ml) to give the product **4** as a dark green solid (142 mg, 0.17 mmol, 84%). **mp.** = 175 – 180 °C (dec.).**^{1}H NMR** (500 MHz, $CDCl_3$, 298 K): 7.99 (d, $^3J_{HH}$ = 7.8 Hz, 1H), 7.75 (d, $^3J_{HH}$ = 7.4 Hz, 1H), 7.53 (t, $^3J_{HH}$ = 7.7 Hz, 1H), 7.45 (br. d, $^3J_{HH}$ = 7.7 Hz, 1H), 7.40 (t, $^3J_{HH}$ = 7.8 Hz, 1H), 7.29 (br. d, $^3J_{HH}$ = 7.6 Hz, 1H), 7.21 – 7.24 (m, 2H), 7.17 – 7.18 (m, 3H), 7.14 (t, $^3J_{HH}$ = 7.5 Hz, 1H), 7.03 (t, $^3J_{HH}$ = 7.1 Hz, 1H), 4.91 (s, 3H). **^{13}C{^{1}H} NMR** (126 MHz, $CDCl_3$, 298 K): 175.0 (s), 144.9 (s), 140.7 (s), 140.0 (s), 139.8 (s), 137.6 (s), 136.7 (s), 135.0 (s), 134.2 (s), 132.3 (s), 132.1 (s), 131.8 (s), 130.6 (s), 128.6 (s), 128.5 (s), 128.2 (s), 127.4 (s), 127.3 (s), 127.1 (s), 126.2 (s), 126.1 (s), 125.5 (s), 117.4 (s), 63.4 (s). Note: carbon atoms of C_6F_5 groups not reported due to extensive line broadening. **^{11}B NMR** (160 MHz, $CDCl_3$, 298 K): -15.8 (s). **^{19}F NMR** (283 MHz, $CDCl_3$, 298 K): -123.03 (br. s, 1F, *o*-F), -125.19 (br. d, $^3J_{FF}$ = 20.3 Hz, 1F, *o*-F), -129.18 (br. d, $^3J_{FF}$ = 20.3 Hz, 1F, *o*-F), -129.62 (br. s, 1F, *o*-F), -131.98 (br. m, 1F, *o*-F), -136.14 (br. s, 1F, *o*-F), -160.78 (t, $^3J_{FF}$ = 20.7 Hz, 1F, *p*-F), -161.72 (t, $^3J_{FF}$ = 20.8 Hz, 1F, *p*-F), -162.68 (t, $^3J_{FF}$ = 20.8 Hz, 1F, *p*-F), -164.87 (t, $^3J_{FF}$ = 24.2 Hz, 1F, *m*-F), -165.49 (m, 1F, *m*-F), -166.58 (m, 4F, *m*-F). **HRMS** (ES^+) *m/z* calculated $[C_{42}H_{16}BF_{15}O_2]^+$ $[M]^+$: 848.1004; observed: 848.1016. **Elemental analysis** (%) calculated, C: 59.49, H: 1.90. Found, C: 59.41, H: 1.92.

2. NMR spectra

2.1 NMR spectra of starting materials

^{1}H NMR (500 MHz, $CDCl_3$, 298 K) spectrum of methyl 2-((trimethylsilyl)ethynyl)benzoate:

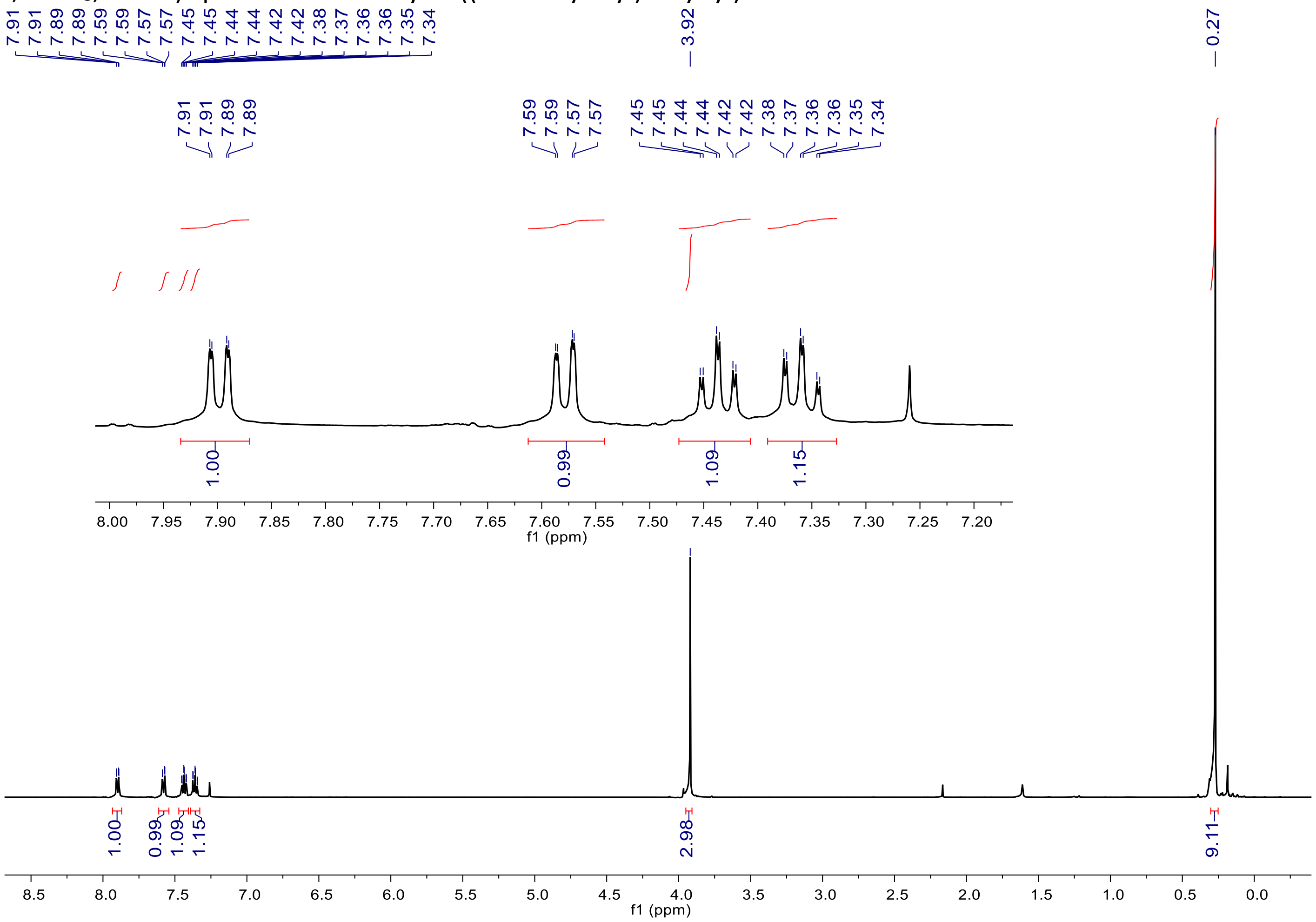

^{1}H NMR (500 MHz, $CDCl_3$, 298 K) spectrum of methyl 2-ethynylbenzoate:

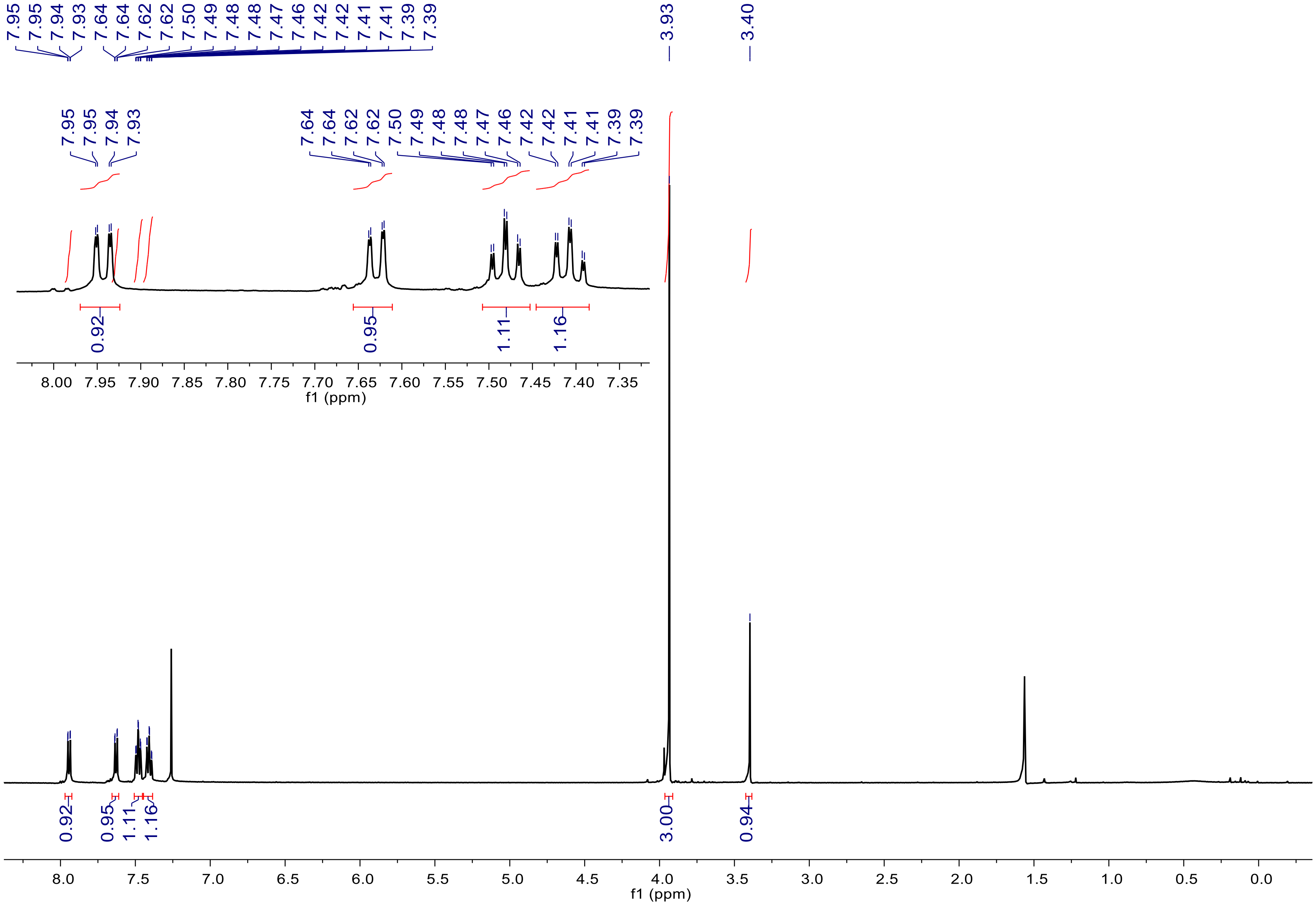

^{1}H NMR (500 MHz, $CDCl_3$, 298 K) spectrum of 1-bromo-2-(phenylethynyl)benzene:

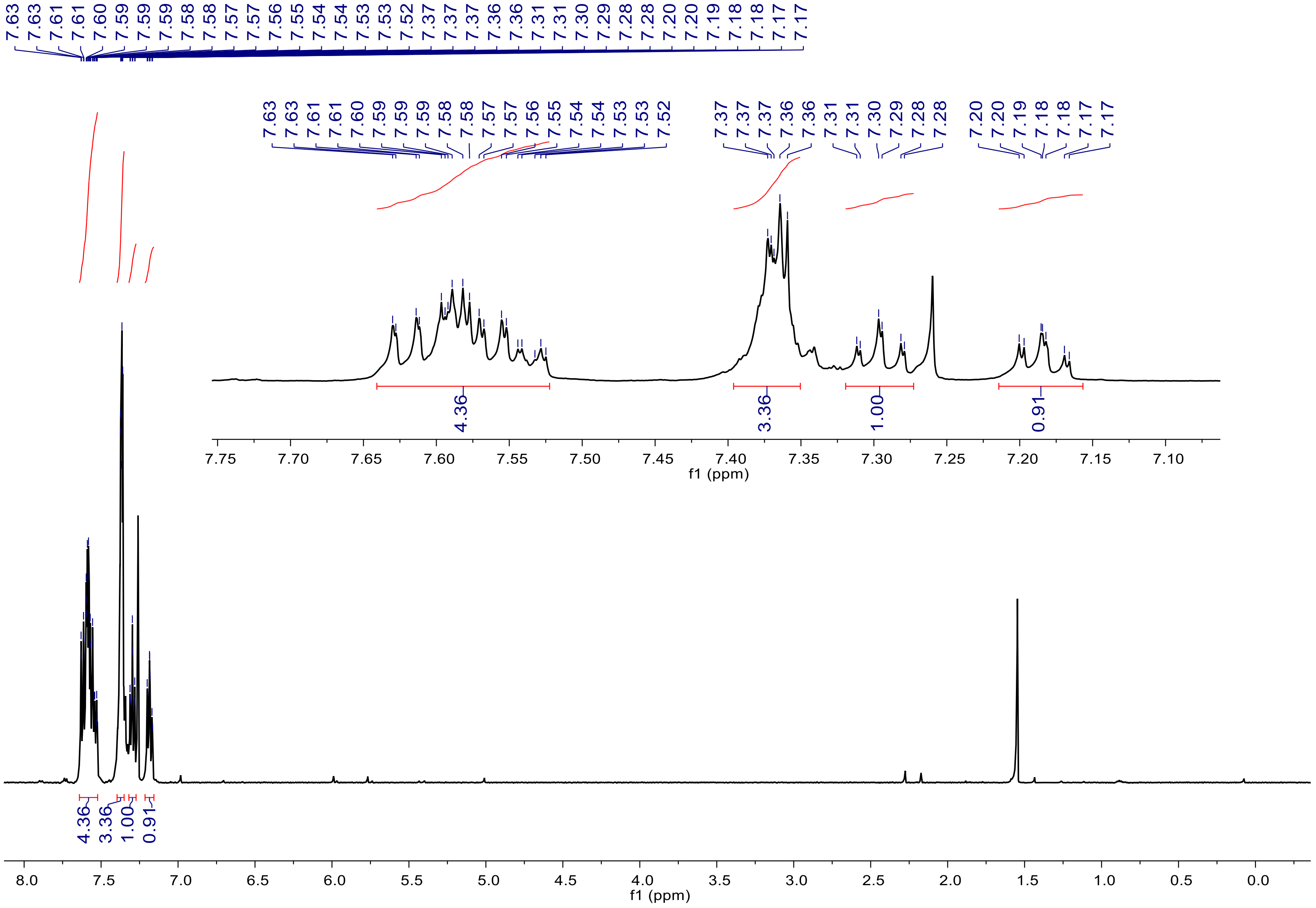

^{1}H NMR (500 MHz, $CDCl_3$, 298 K) spectrum of methyl-2-((2-(phenylethynyl)phenyl)ethynyl)benzoate (**1**):

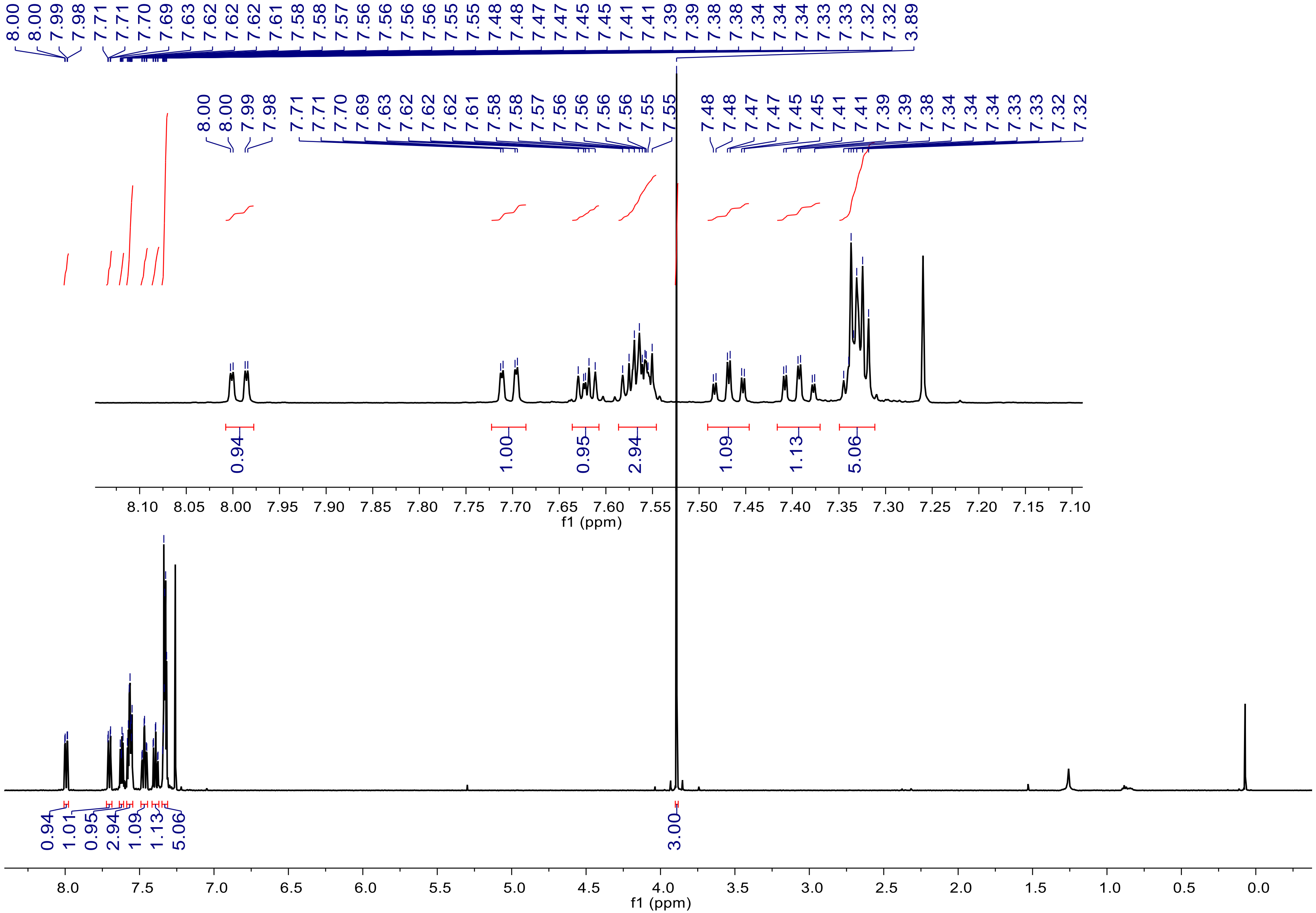

2.2 NMR spectra of products

^{1}H NMR (400 MHz, CD_2Cl_2, 298 K) spectrum of **2**:

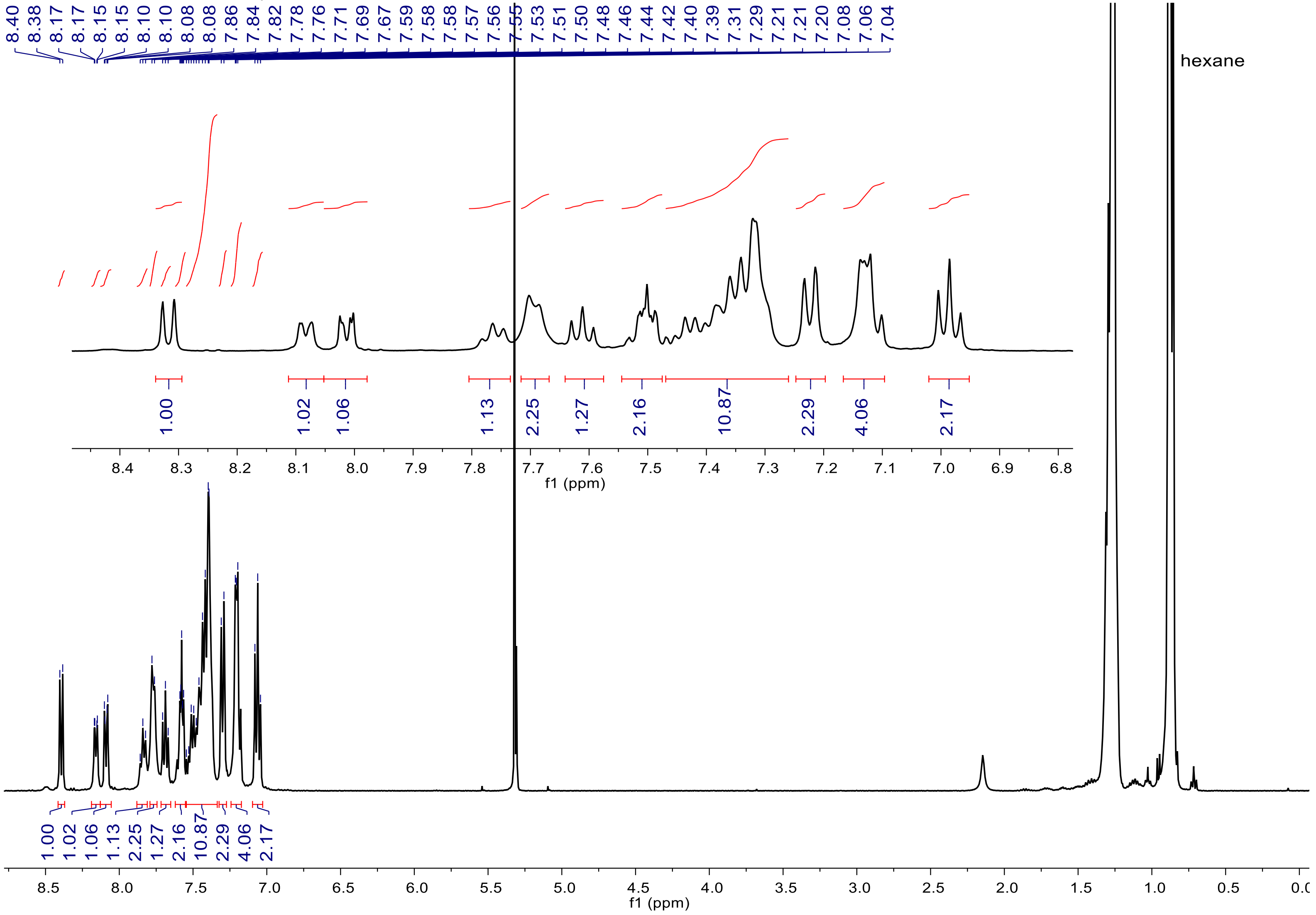

$^{13}C\{^{1}H\}$ NMR (101 MHz, CD_2Cl_2, 298 K) spectrum of **2**:

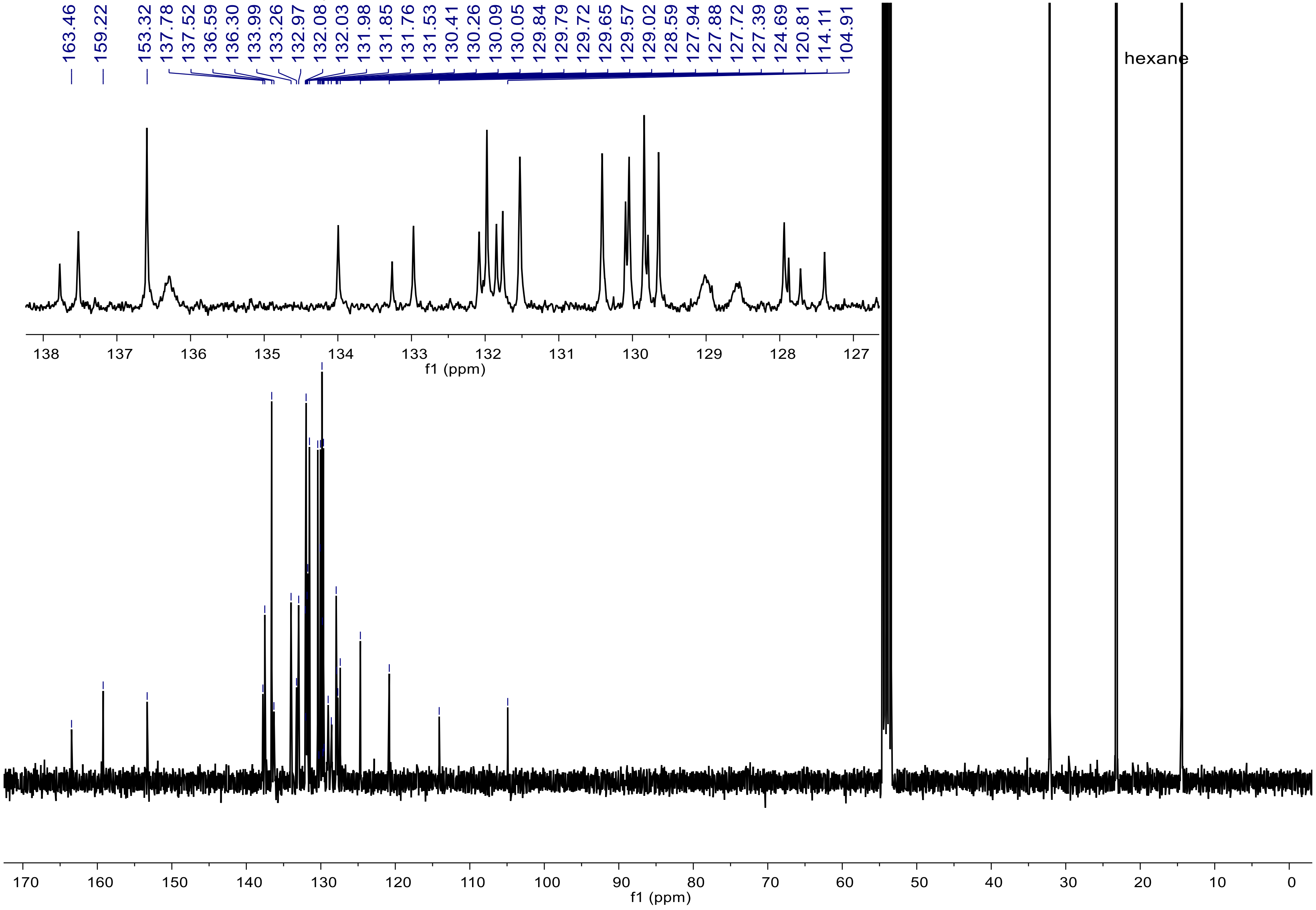

^{1}H NMR (500 MHz, $CDCl_3$, 298 K) spectrum of **3a**:

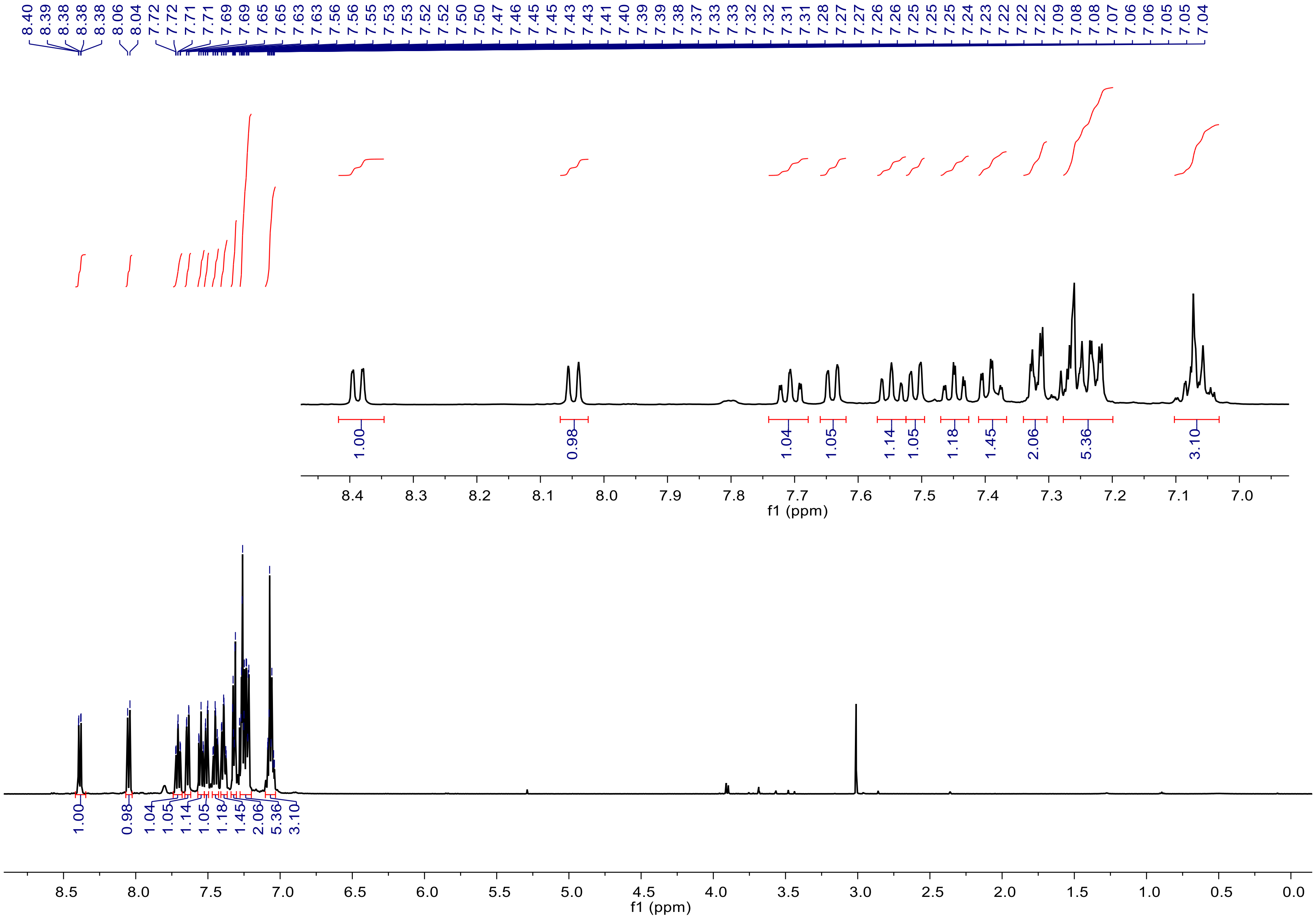

$^{13}C\{^1H\}$ NMR (126 MHz, $CDCl_3$, 298 K) spectrum of **3a**:

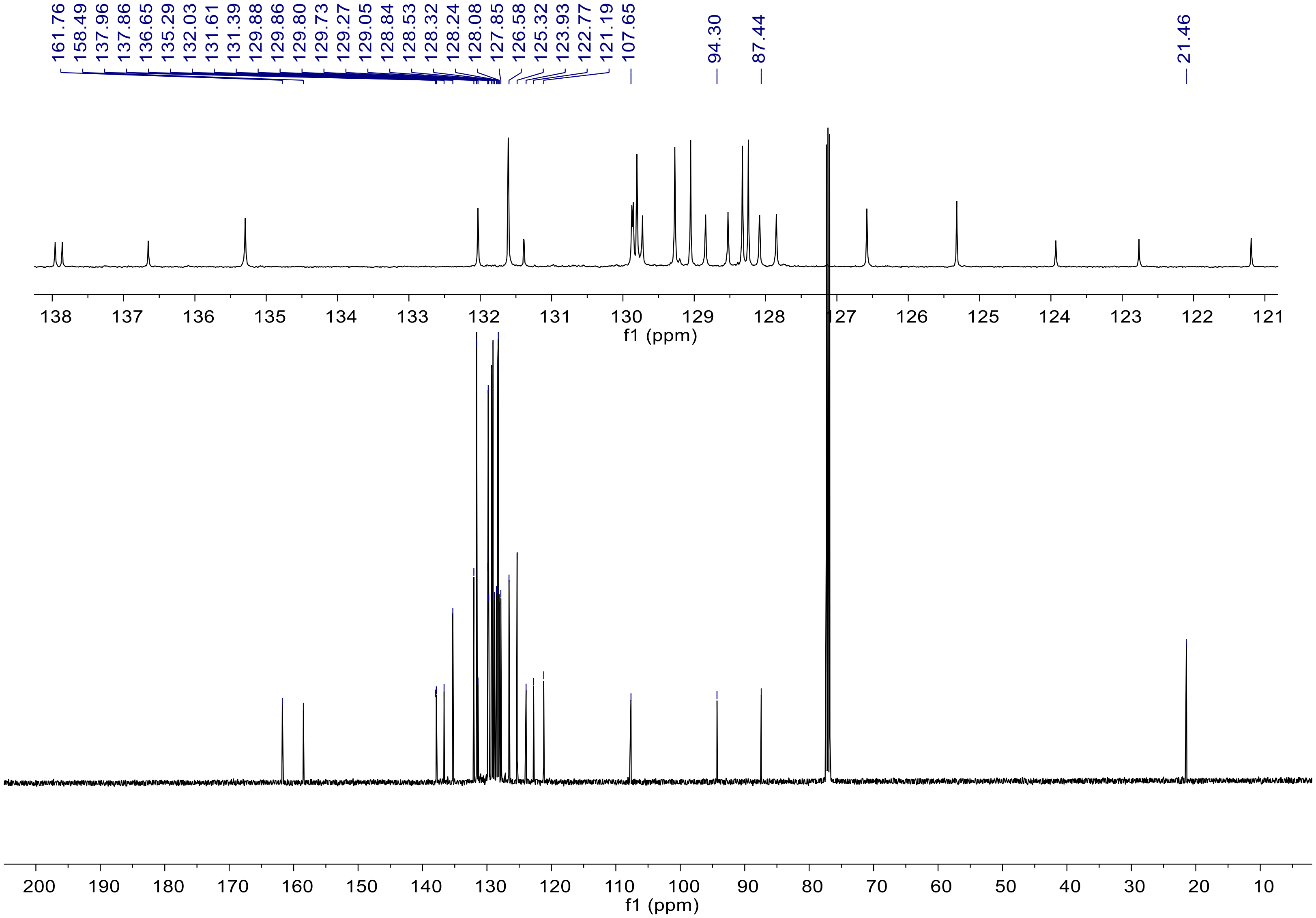

^{77}Se NMR (95 MHz, $CDCl_3$, 298 K) spectrum of **3a**:

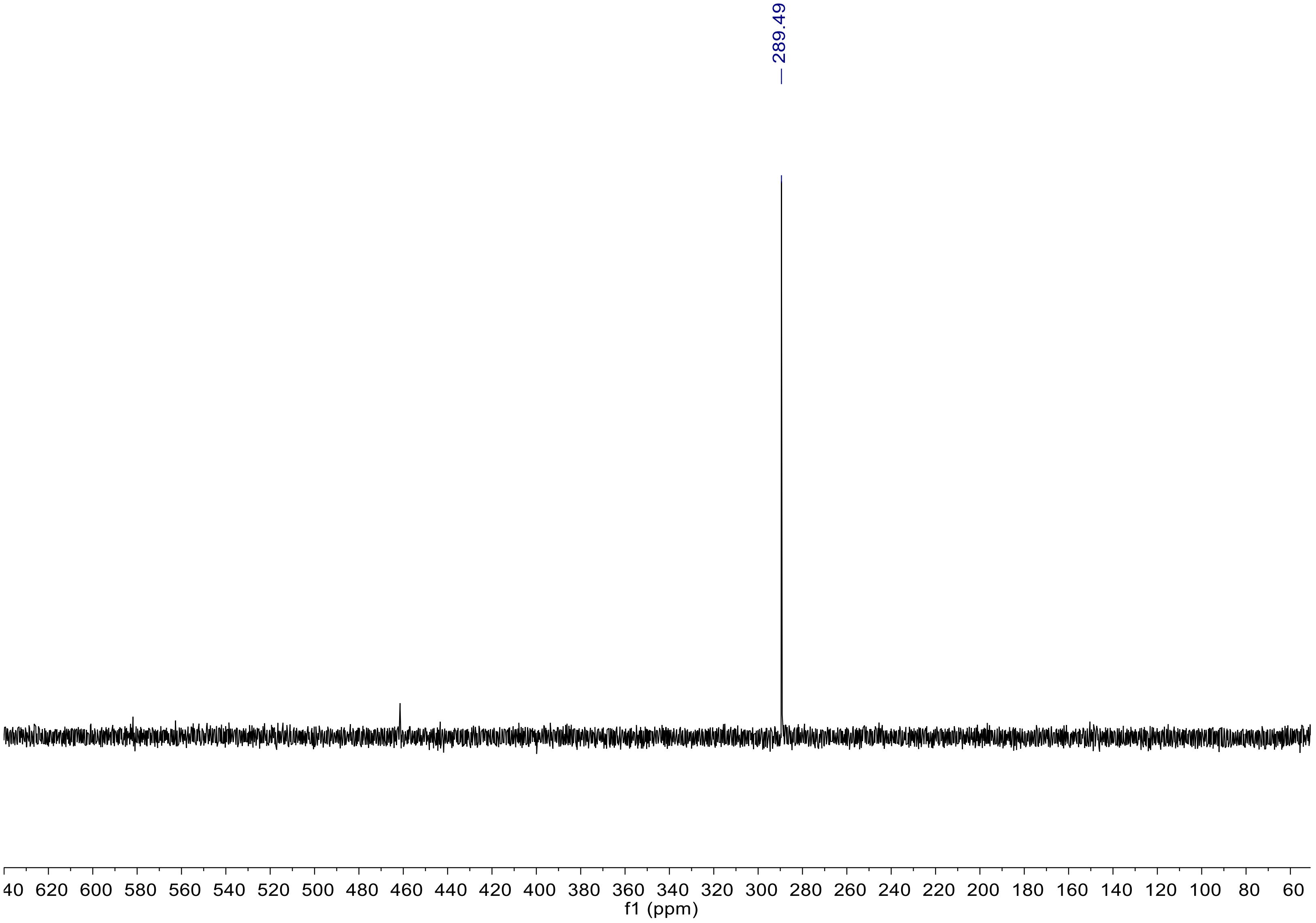

^{1}H NMR (500 MHz, $CDCl_3$, 298 K) spectrum of **3b**:

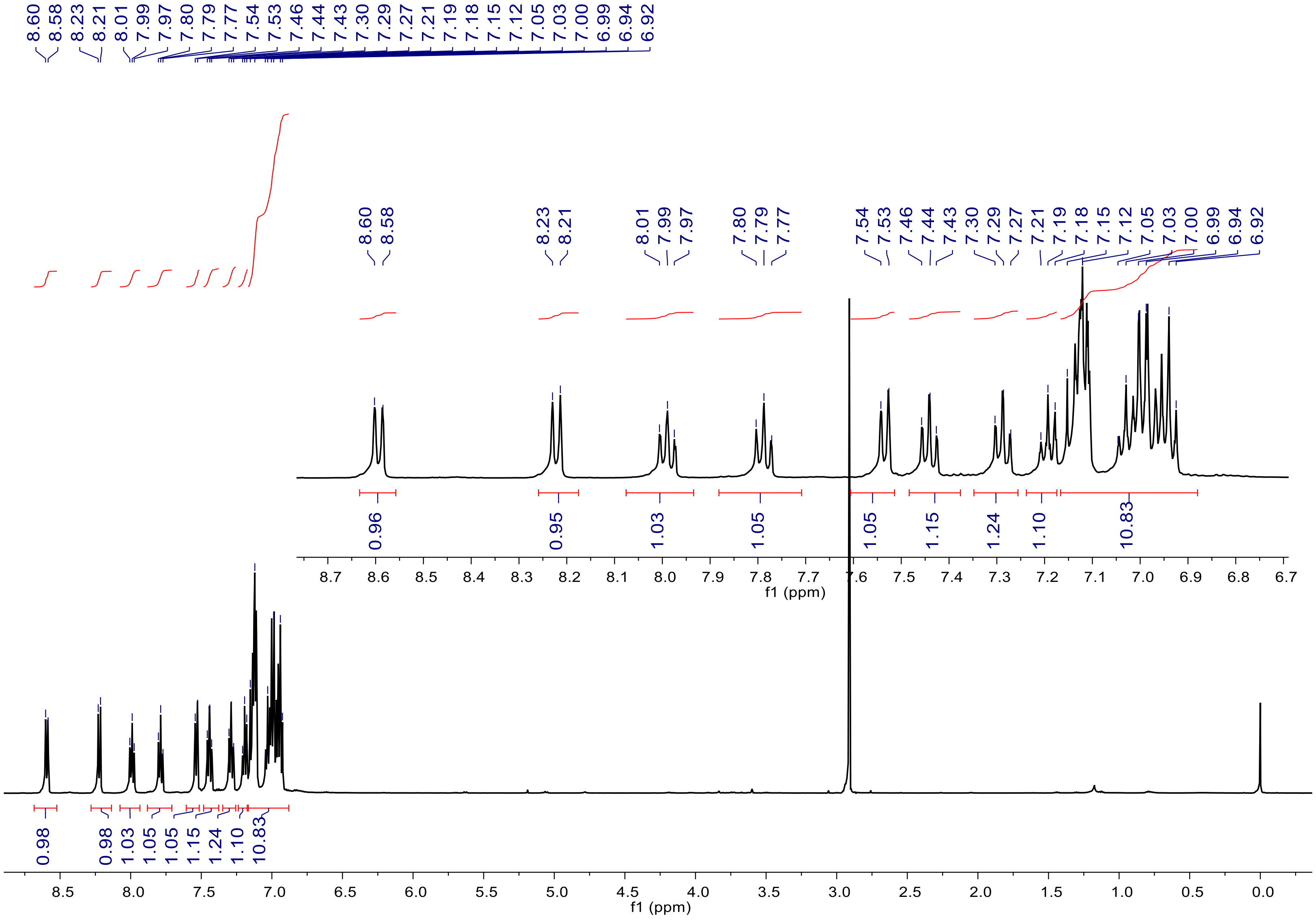

$^{13}C\{^{1}H\}$ NMR (126 MHz, $CDCl_3$, 298 K) spectrum of **3b**:

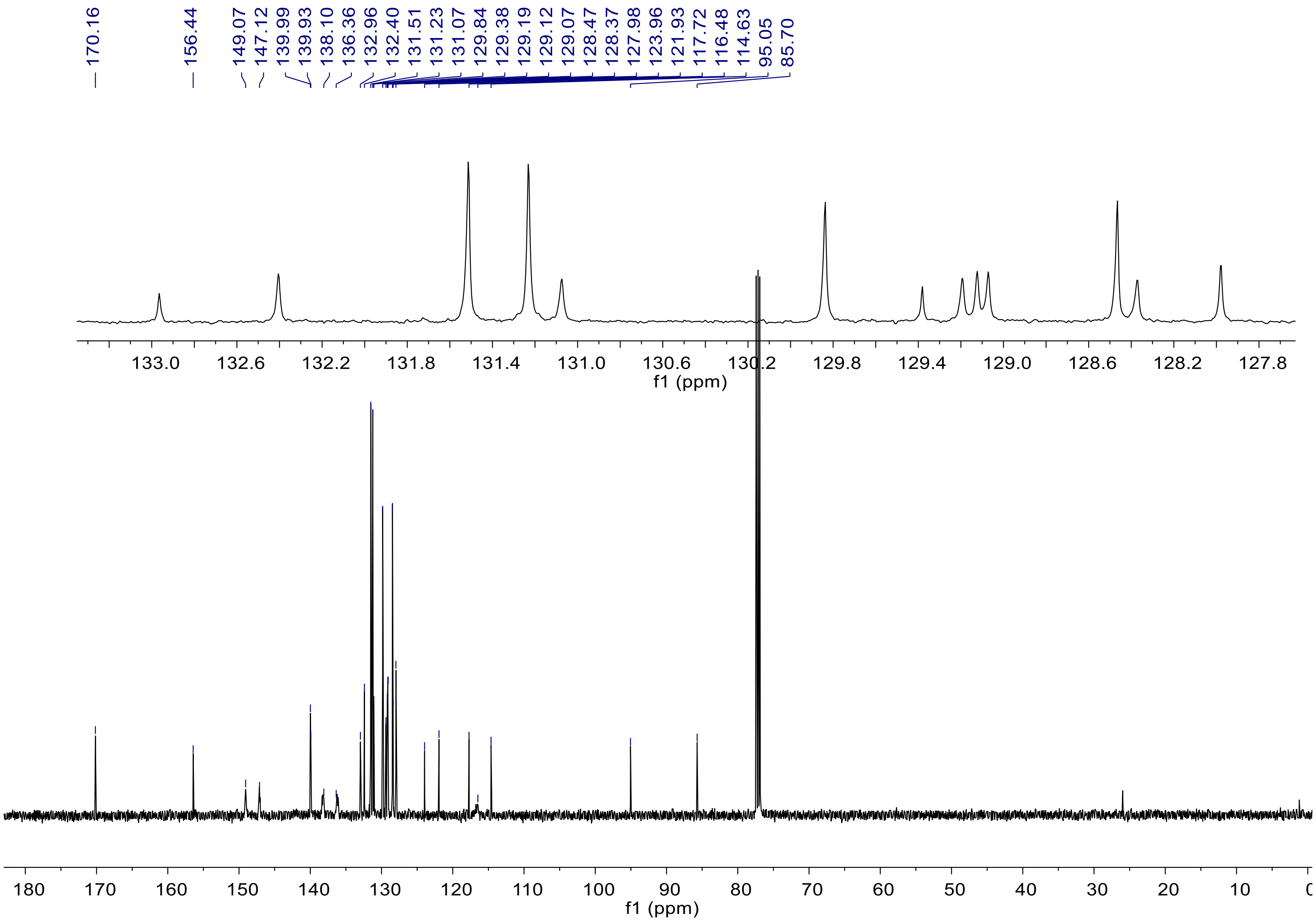

^{77}Se NMR (95 MHz, $CDCl_3$, 298 K) spectrum of **3b**:

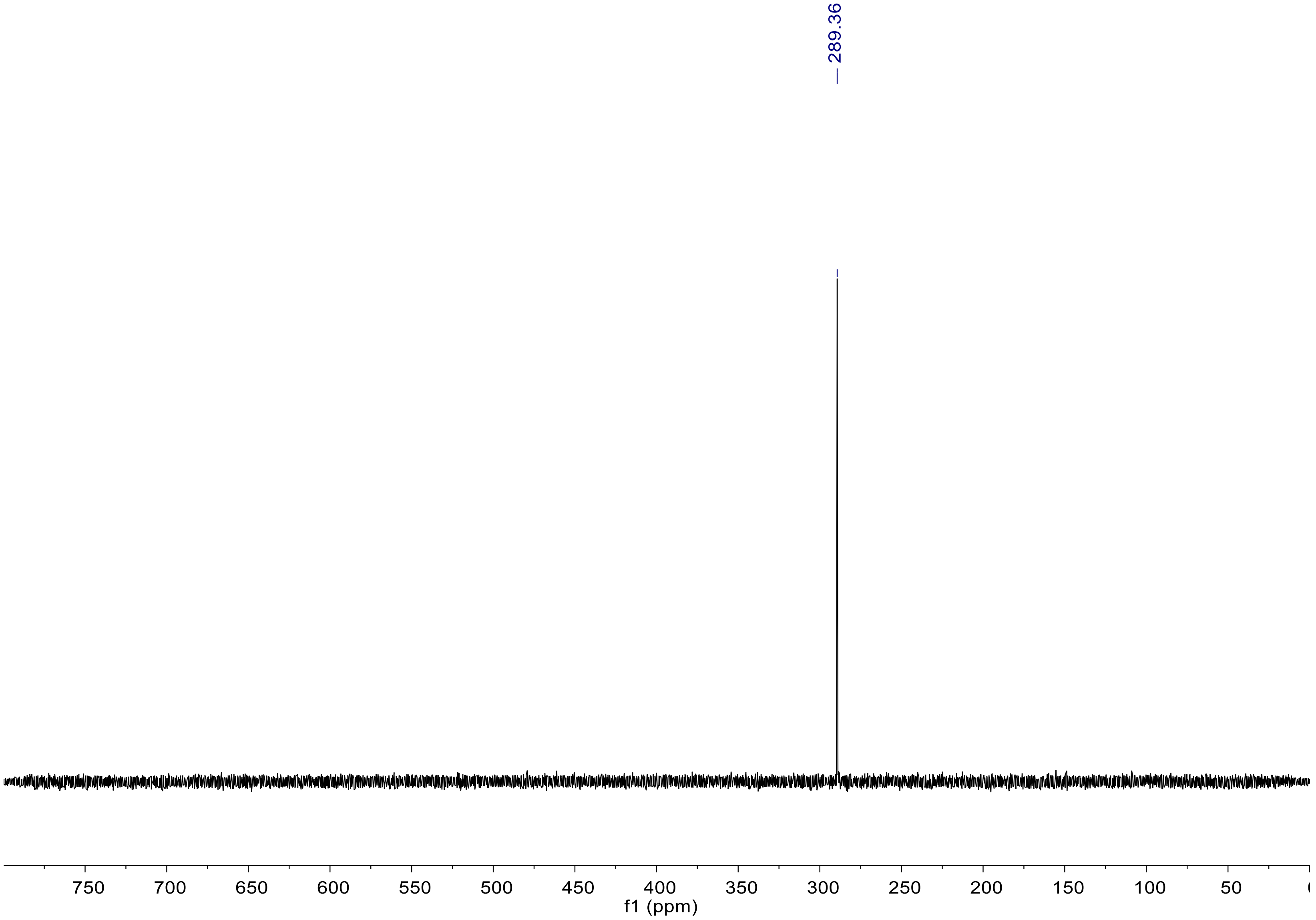

^{19}F NMR (283 MHz, $CDCl_3$, 298 K) spectrum of **3b**:

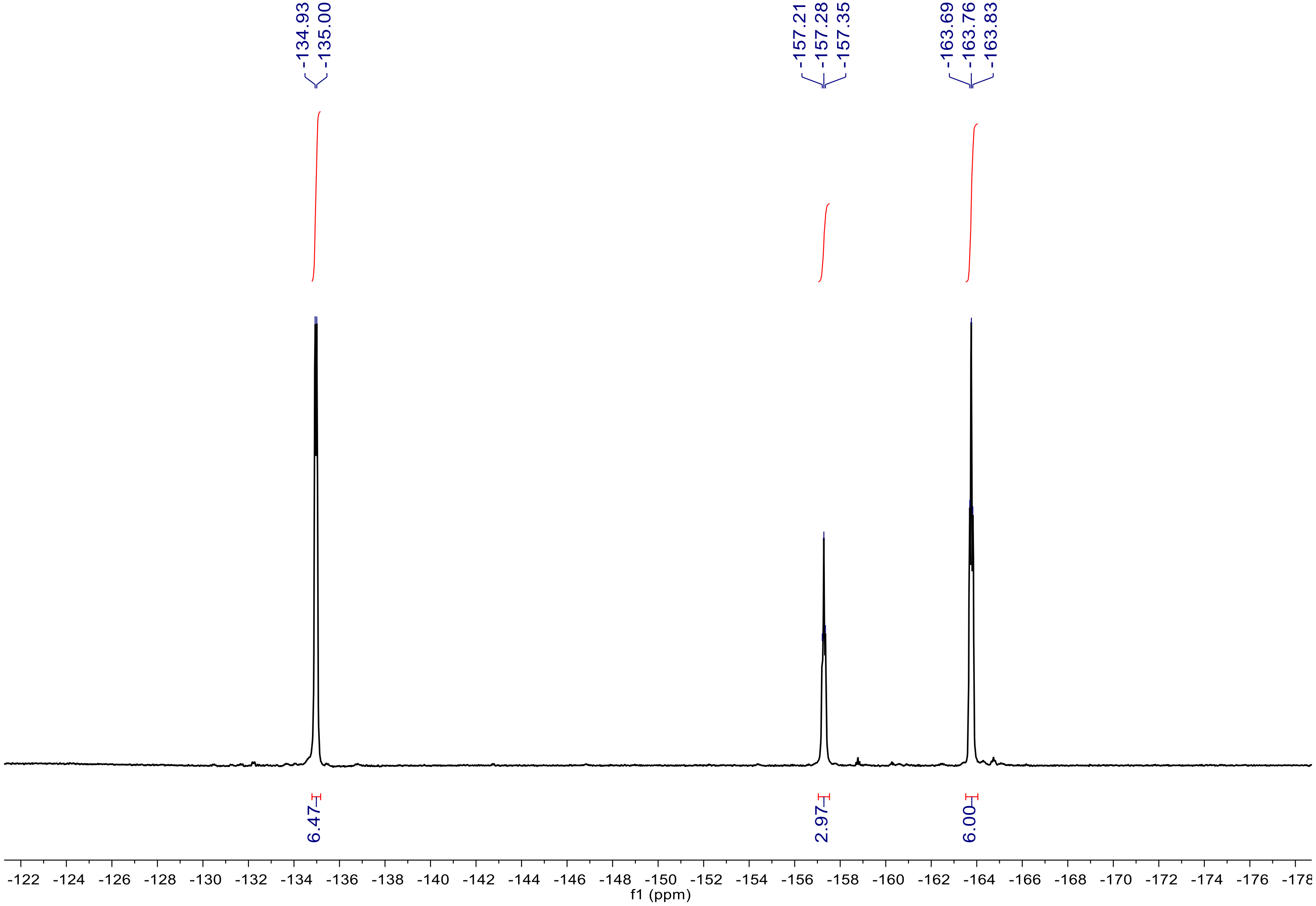

^{11}B NMR (160 MHz, $CDCl_3$, 298 K) spectrum of **3b**:

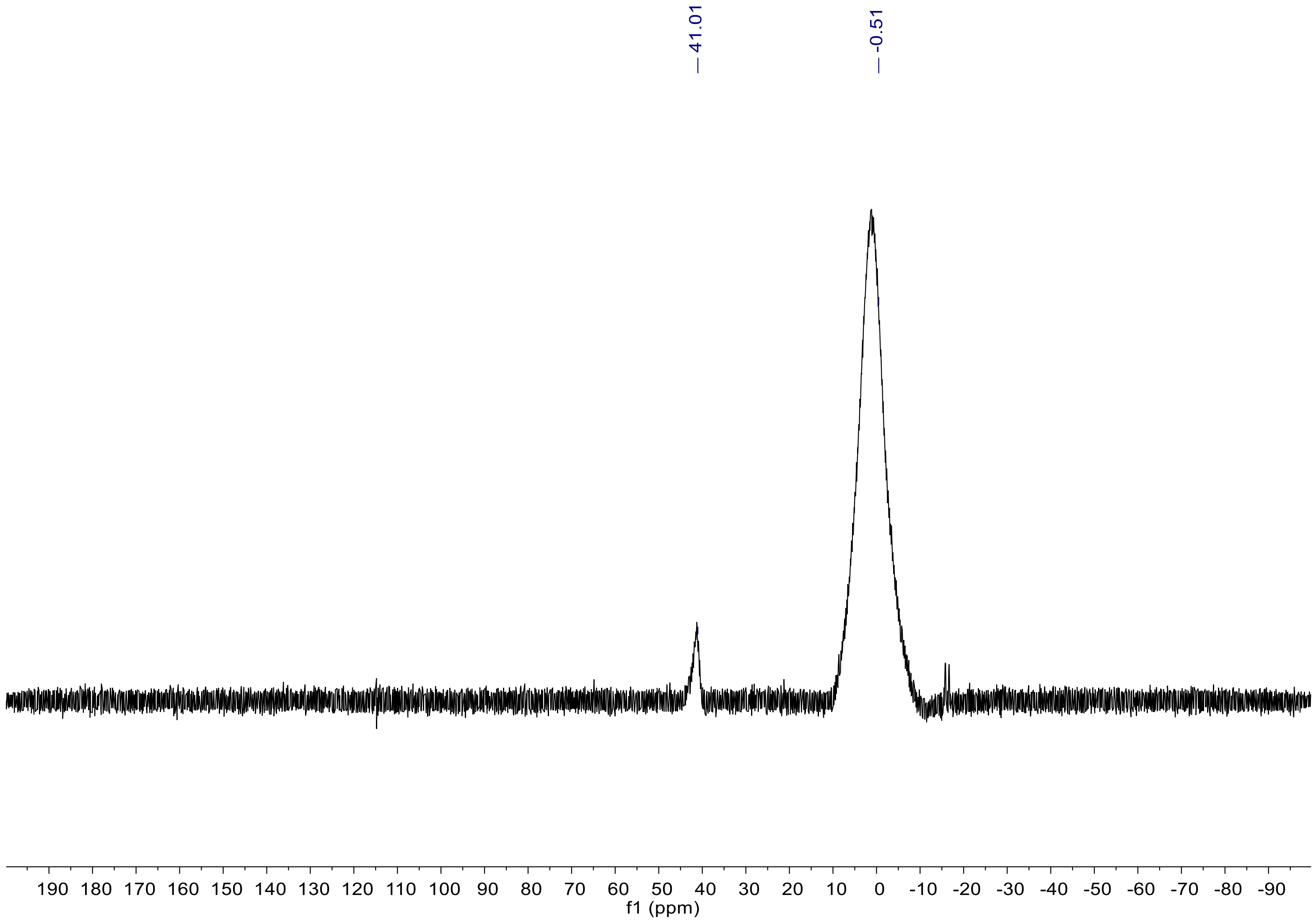

Stacked ^{1}H spectra of **1** (bottom), **3a** (middle), **3b** (top):

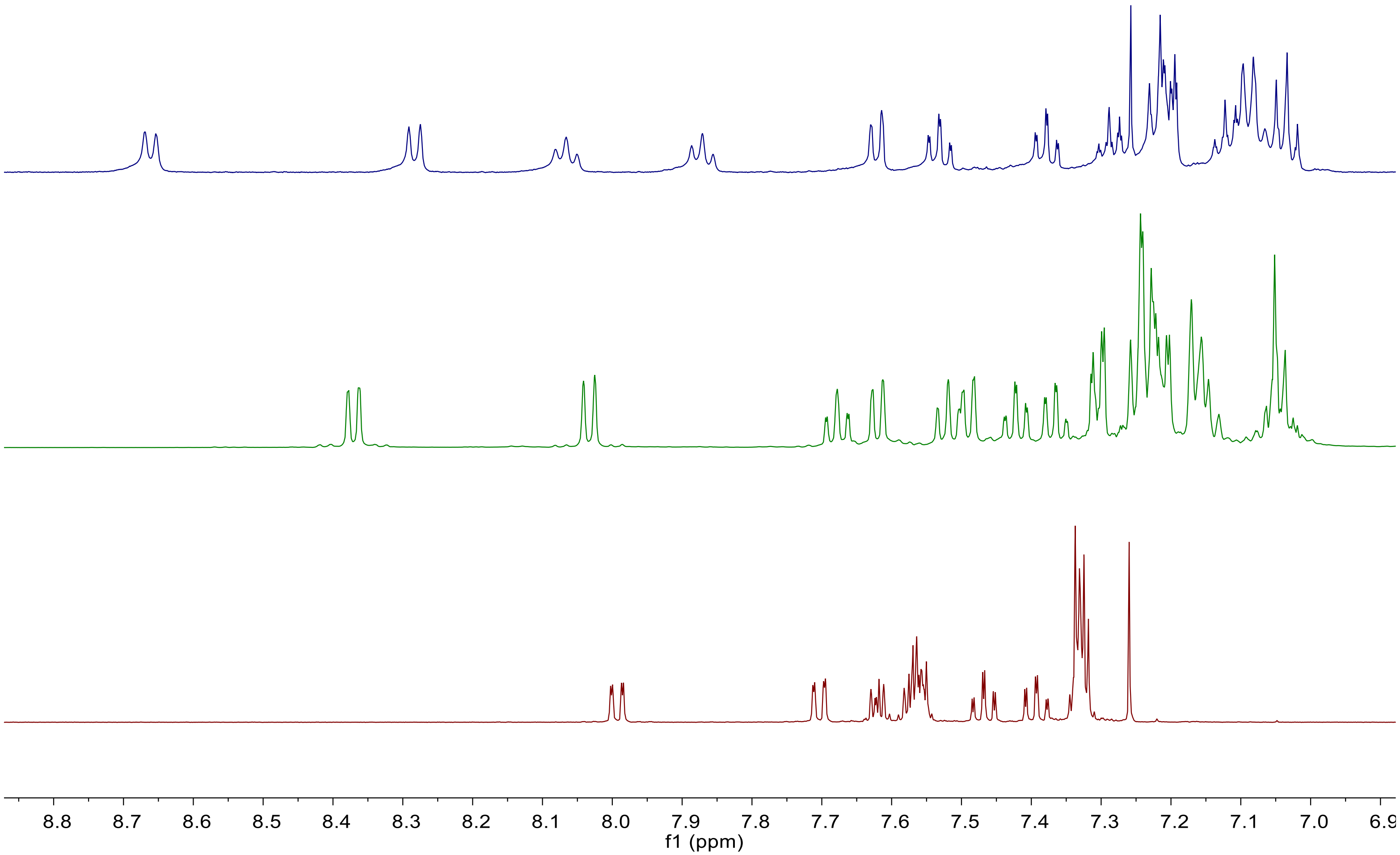

^1H NMR (500 MHz, $CDCl_3$, 298 K) spectrum of **4**:

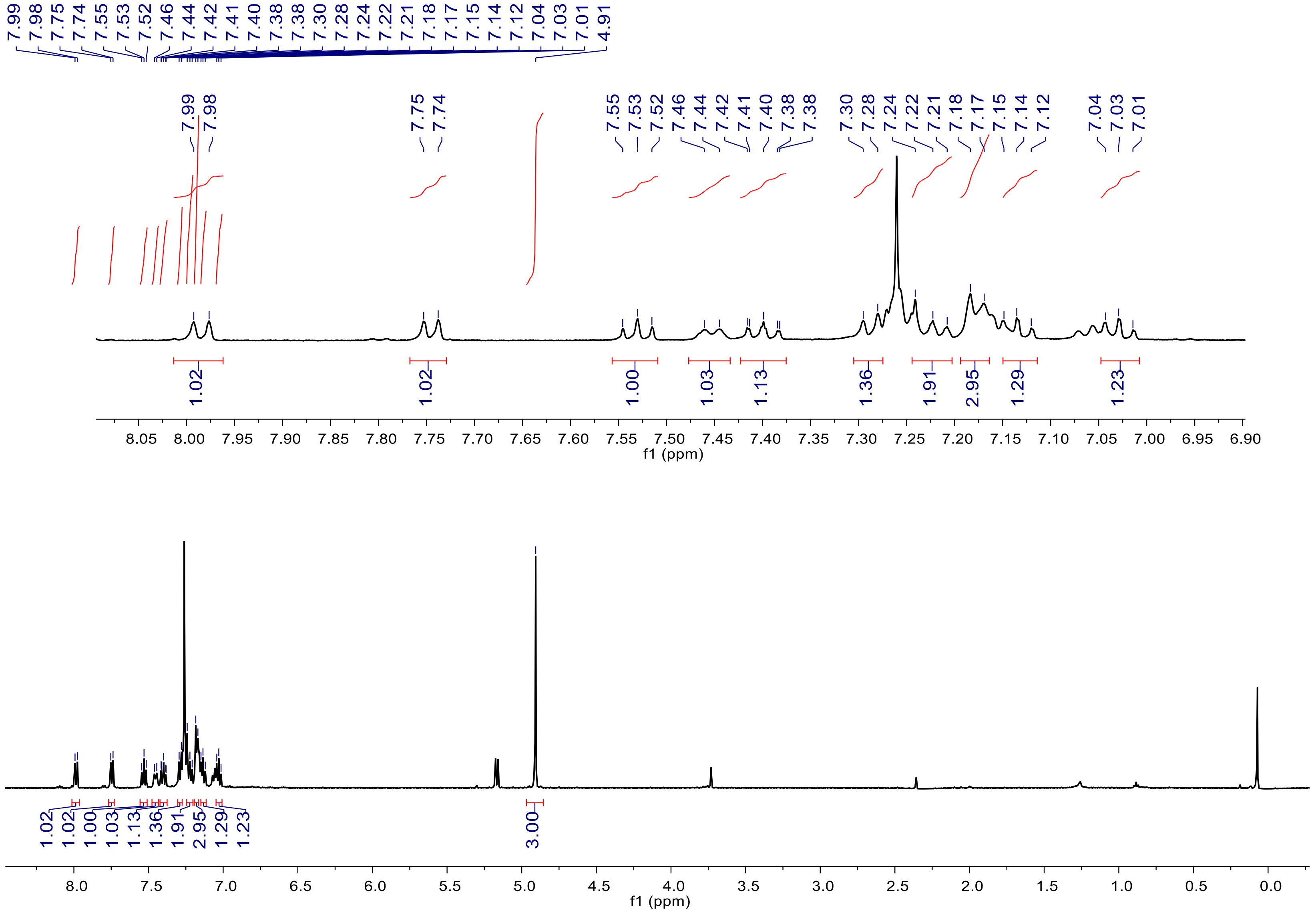

$^{13}C\{^{1}H\}$ NMR (126 MHz, $CDCl_3$, 298 K) spectrum of **4**:

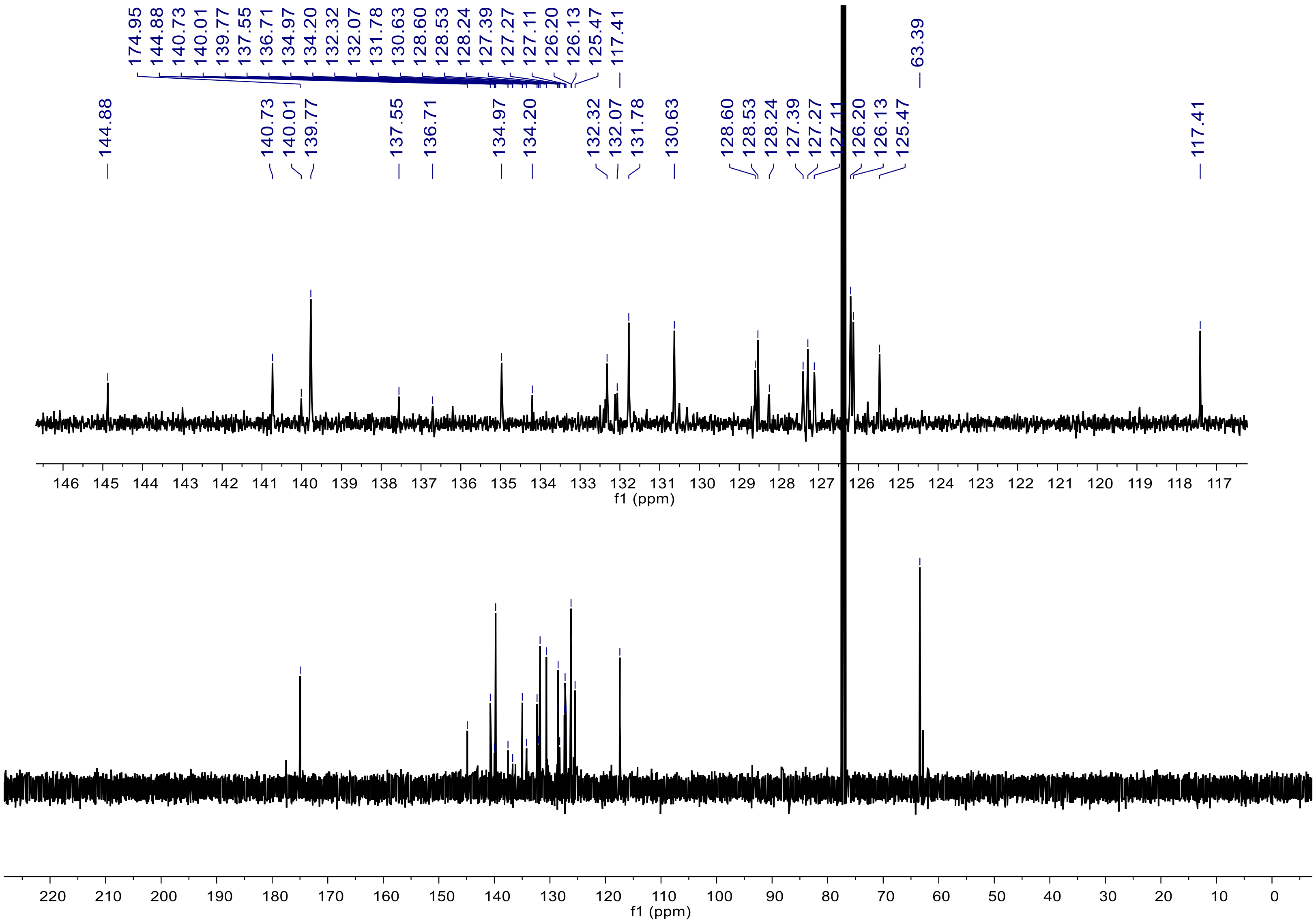

^{19}F NMR (283 MHz, $CDCl_3$, 298 K) spectrum of **4:**

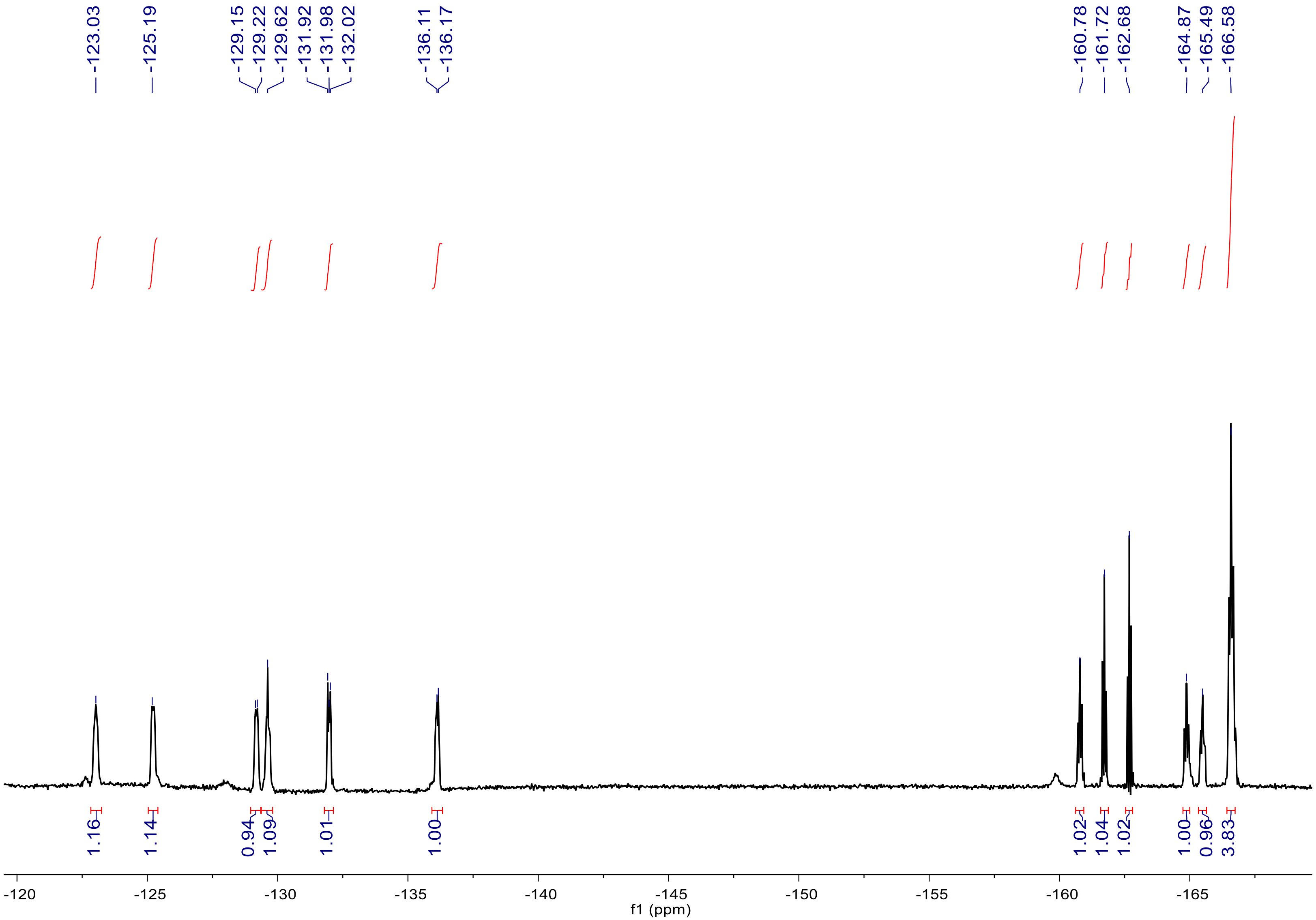

^{11}B NMR (160 MHz, $CDCl_3$, 298 K) spectrum of **4**:

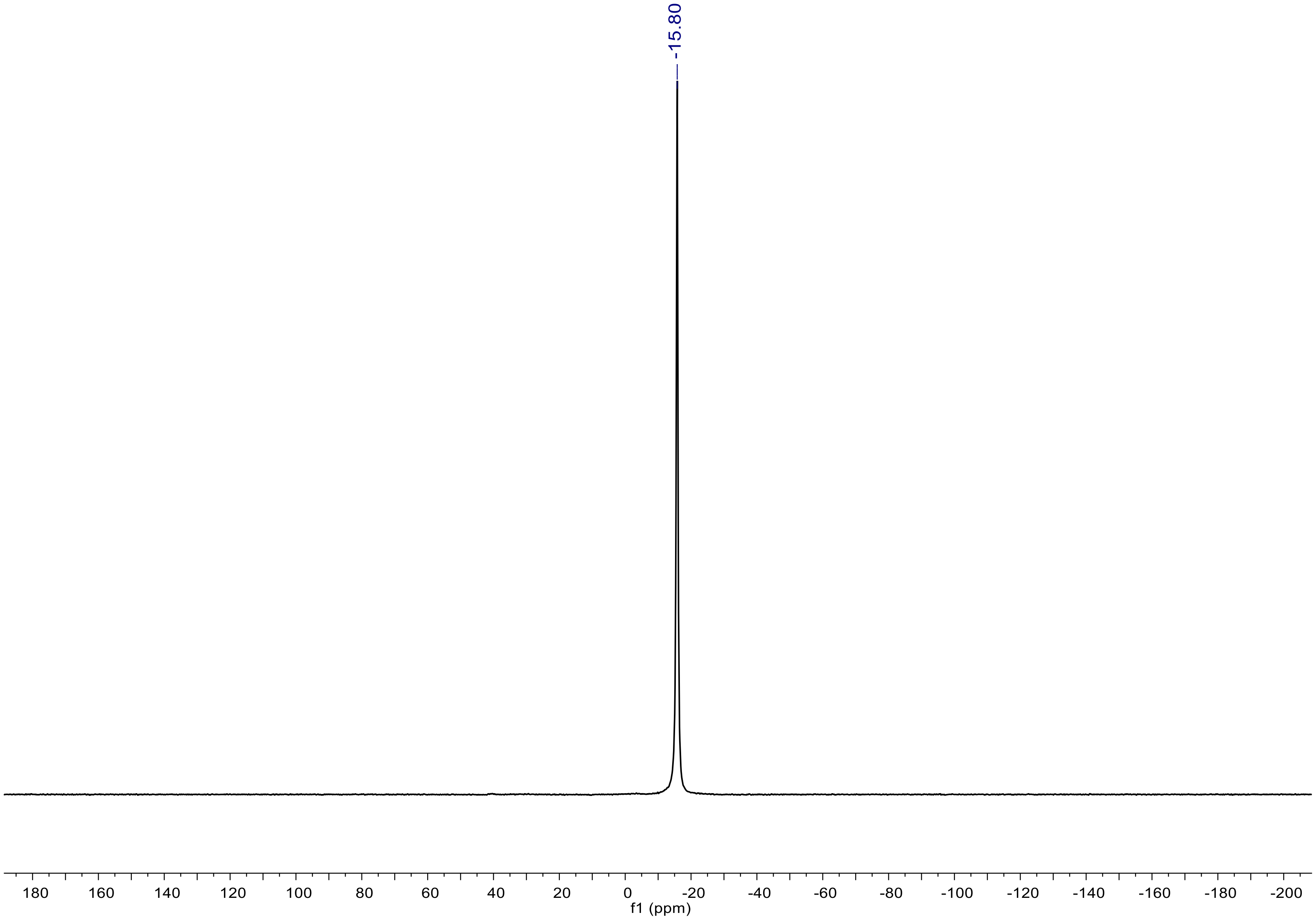

3. X-ray crystallography

Single crystals of **2**, **3b** and **4** were grown under an inert atmosphere of N_2. Crystallographic studies were undertaken of a single crystal mounted in paratone and studied on an Agilent SuperNova Dual Atlas three-circle diffractometer using Mo-Kα or Cu- Kα radiation and a CCD detector. Measurements were carried out at 150(2) K with temperatures maintained using an Oxford cryostream. Data were collected and integrated and data corrected for absorption using a numerical absorption correction based on gaussian integration over a multifaceted crystal model within CrysAlisPro.[2] The structures were solved by direct methods and refined against F^2 within SHELXL-2013.[3] A summary of crystallographic data are available as ESI and the structures deposited with the Cambridge Structural Database (CCDC deposition numbers 1480722 – 1480724). These data can be obtained free of charge from The Cambridge Crystallographic Data Centre *via* www.ccdc.cam.ac.uk/data_request/cif

3.1 Table of crystallographic details

Table 1 Geometric parameters for Compounds **2**, **3b** and **4**.

**Compound**	**2**	**3b**	**4**
**Empirical Formula**	$C_{42}H_{29}Cl_5O_2Se_3$	$C_{53}H_{32}BF_{15}O_2Se$	$C_{49}H_{24}BF_{15}O_2$
**Crystal System**	*Triclinic*	*Monoclinic*	*Monoclinic*
**Space Group**	*P-1*	*P 2_1/c*	*P 2_1/c*
***a*/Å**	9.1231(4)	17.4913(9)	15.9561(6)
***b*/Å**	13.3695(6)	12.8840(7)	18.8796(7)
***c*/Å**	16.6564(8)	20.4750(10)	13.6604(7)
**α/°**	73.574(4)	90	90
**β/°**	83.219(4)	96.260(4)	102.743(4)
**γ/°**	87.728(4)	90	90
**V/Å³**	1934.99(15)	4586.7(4)	4013.8(3)
Z	2	4	4
***T* /K**	150(2)	150(2)	150(2)
**D_c/g.cm^{-3}**	1.682	1.558	1.556
**Crystal size/mm**	0.360 x 0.110 x 0.030	0.343 x 0.151 x 0.051	0.320 x 0.120 x 0.050
**Total data**	13349	25867	13686
**Unique data**	7599	8046	6802
**R_{int}**	0.0458	0.0663	0.0337
**$R_1[F^2>2\sigma F^2)]$**	0.0357	0.0558	0.0500
**wR2 (all data)**	0.1002	0.1367	0.1498
**GoF**	1.036	1.054	1.022
**$\theta_{min}/\theta_{max}$/eÅ^{-3}**	-0.601/0.850	-0.794/0.880	-0.243/0.484
CCDC code	1480724	1480723	1480722

3.2 Crystal structures

3.2.1 Solid-state structure of **2** with 50% probability ellipsoids. C: black, O: red, H: white, Se: Orange, Cl: aqua-marine. $CHCl_3$ solvent molecule removed for clarity.

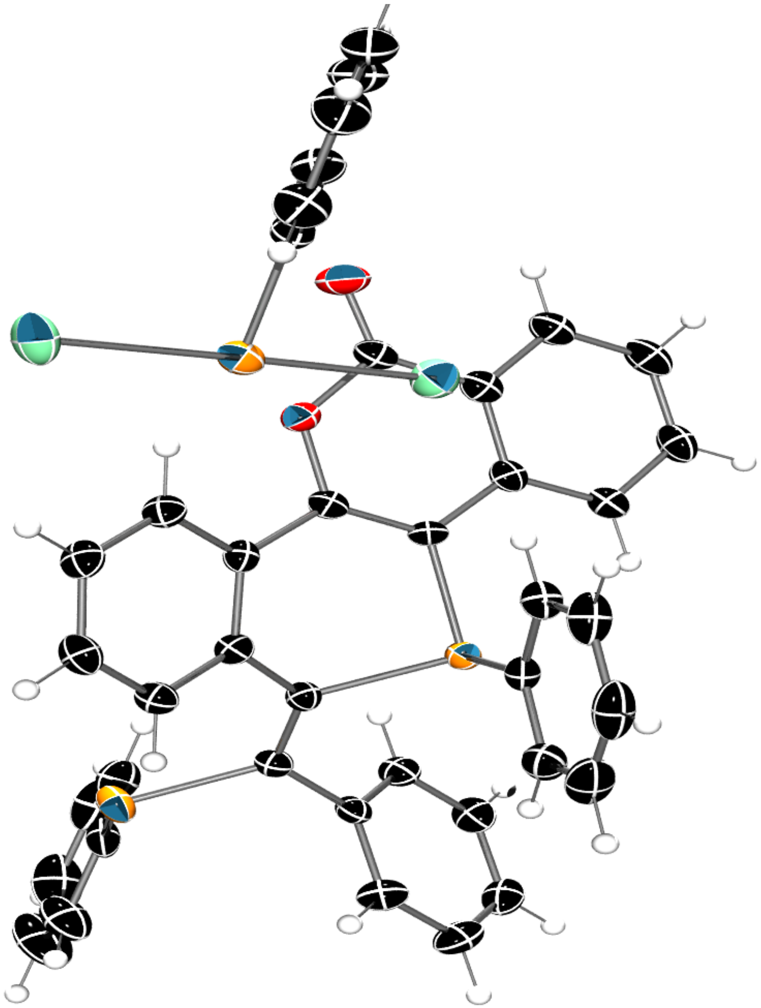

3.2.3 Solid-state structure of **3b** with 50% probability ellipsoids. C: black, O: red, B: yellow-green, F: pink, H: white, Se: orange.

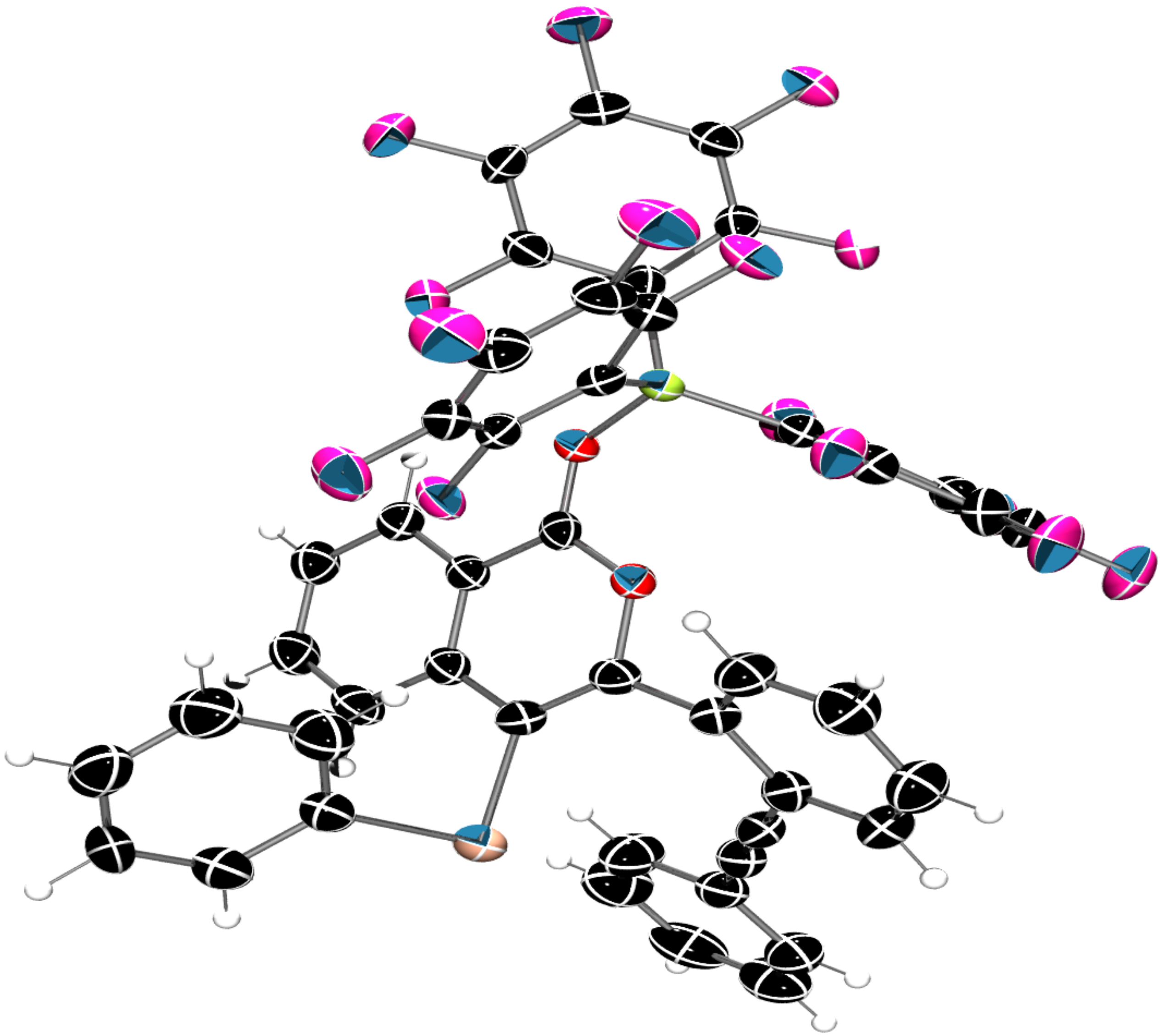

3.2.4 Solid-state structure of **4** with 50% probability ellipsoids. C: black, O: red, B: yellow-green, F: pink, H: white. Toluene solvent molecule omitted for clarity.

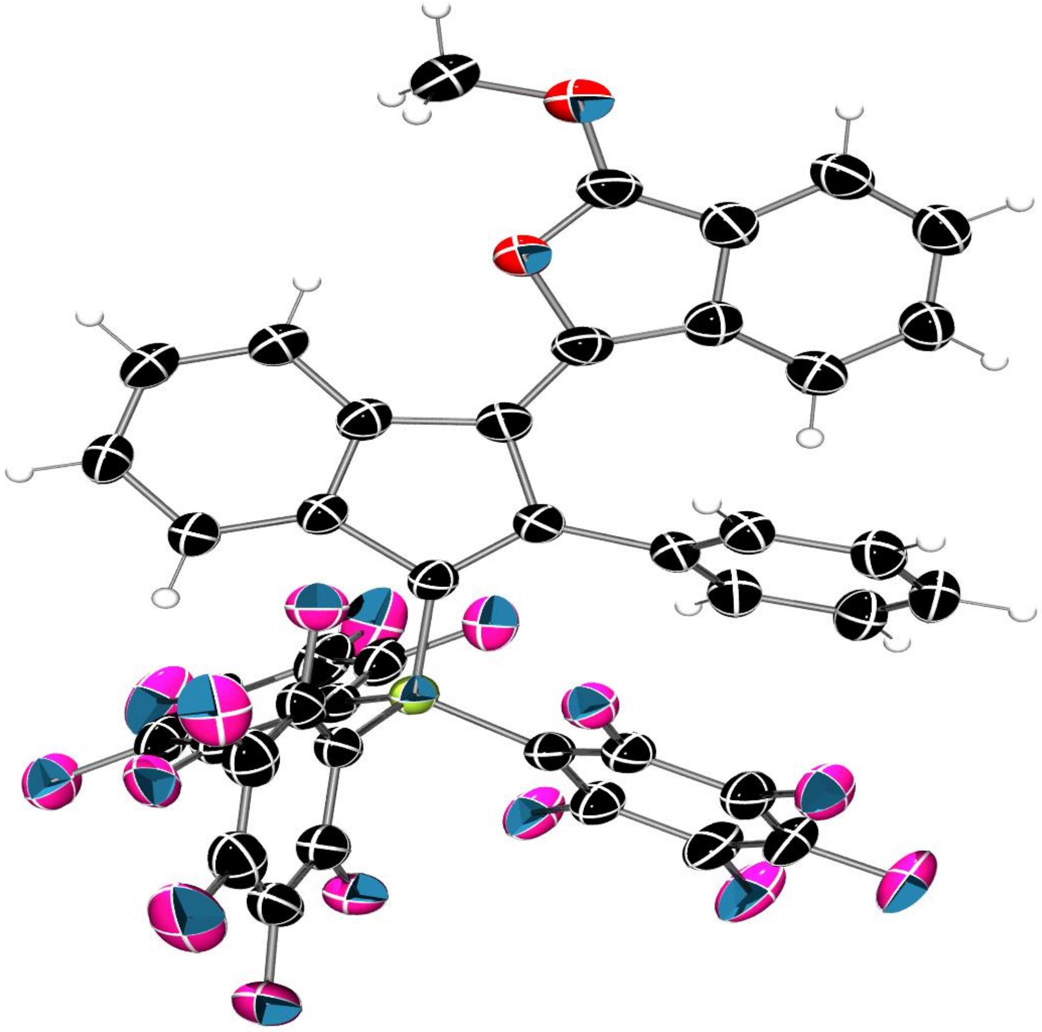

3.2.2 Solid-state space filling structure of **4**. C: black, O: red, B: yellow-green, F: pink, H: white. Toluene solvent molecule omitted for clarity.

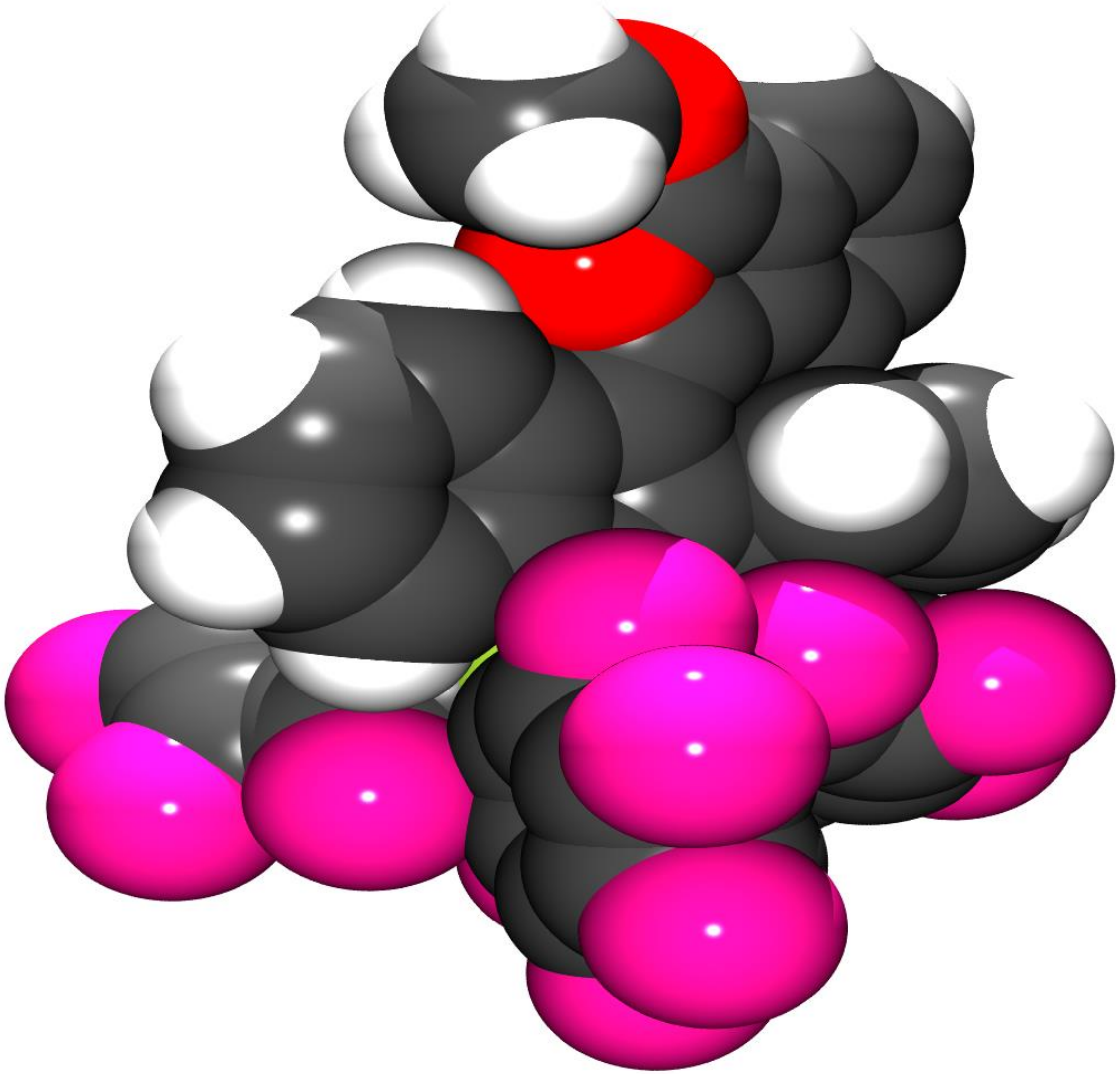

4. References

1. Z. Lia, J. Honga, L. Wenga, X. Zhou, *Tetrahedron*, **2012**, *68*, 1552.
2. CrysAlisPro, Agilent Technologies, Version 1.171.37.33 (release 27-03-2014 CrysAlis171 .NET).
3. SHELXL-2013, G. M. Sheldrick, University of Göttingen, Germany (2013).
